# Restoring cortical disinhibition improves Huntington’s disease phenotypes

**DOI:** 10.1038/s41586-026-10671-9

**Published:** 2026-07-01

**Authors:** Sonja Blumenstock, David Arakelyan, Nicholas del Grosso, Sonja Schneider, Yufeng Shao, Enida Gjoni, Rüdiger Klein, Irina Dudanova, Takaki Komiyama

**Affiliations:** 1 Department of Neurobiology, Center for Neural Circuits and Behavior, University of California San Diego, La Jolla, CA USA; 2 Department of Molecules–Signaling–Development, Max Planck Institute for Biological Intelligence, Martinsried, Germany; 3 Molecular Neurodegeneration Group, Max Planck Institute for Biological Intelligence, Martinsried, Germany; 4 Department of Neurosciences, University of California San Diego, La Jolla, CA USA; 5 Center for Anatomy, Faculty of Medicine and University Hospital Cologne, University of Cologne, Cologne, Germany; 6 Cologne Excellence Cluster on Cellular Stress Responses in Aging-Associated Diseases (CECAD), University of Cologne, Cologne, Germany; 7 Institute of Anatomy and Cell Biology, University of Würzburg, Würzburg, Germany; 8 Halıcıoğlu Data Science Institute, University of California San Diego, La Jolla, CA USA

**Keywords:** Huntington's disease, Neural circuits, Motor cortex

## Abstract

Huntington’s disease (HD) is a devastating movement disorder without a cure at present^1^. Although the monogenic basis of HD is well defined^2^, the complex downstream effects that underlie behavioural symptoms are poorly understood. These effects include cortical dysfunction^3,4^, yet the roles of specific cortical neuronal subtypes in HD symptoms remain largely unexplored. Here we used longitudinal in vivo two-photon calcium imaging to examine the activity of three cortical inhibitory neuron (IN) subtypes and excitatory corticostriatal (CStr) projection neurons in the motor cortex of the transgenic R6/2 HD mouse model throughout disease progression. We found that motor deficits in R6/2 mice were accompanied by neuron subtype-specific abnormalities in movement-related activity. This included marked hypoactivity of vasoactive intestinal peptide (VIP)-INs and CStr neurons, which was also observed in the knock-in zQ175DN HD mouse model. Optogenetic activation of VIP-INs in R6/2 mice restored healthy levels of activity in VIP-INs and their downstream CStr neurons and ameliorated motor deficits in R6/2 mice; behavioural improvements persisted for days after stimulation. Our findings highlight cortical INs as a potential therapeutic target for HD.

## Abstract (web summary)

In a mouse model of Huntington’s disease, targeted modulation of neuronal activity restores network function and improves motor deficits.

## Main

Precise motor control is essential for the survival and well-being of an animal. This function is subserved by complex, interconnected neuronal networks in the brain that include the neocortex and striatum^5^. Various neurodegenerative diseases affect motor control, including Huntington’s disease (HD). HD is a devastating monogenic disorder without a cure at present; it is caused by a CAG repeat expansion in exon 1 of the huntingtin (*HTT*) gene^1,2^. The disease is characterized by severe and progressive motor dysfunction. Although the genetic foundation of HD is well understood^2^, the precise neural circuit basis that leads to HD motor symptoms remains unclear. Of note, one of the key brain regions associated with the symptomatology and progression of HD is the neocortex and its projections to the striatum. Changes in corticostriatal connections are among the earliest events in HD progression, and disconnection from cortical afferents probably has a major role in the subsequent dysfunction of the downstream striatal circuits^3,6,7^. Furthermore, cortical inhibitory circuits, which regulate the activity of excitatory corticostriatal (CStr) projection neurons, seem to be affected in HD^8–10^. These observations raise the possibility that HD has cell-type-specific effects on cortical networks. Many neuron types are molecularly identifiable and genetically targetable, affording the potential for subtype-specific therapeutic interventions.

Cortical inhibitory neurons (INs) consist of distinct subtypes with unique functions shaped by their morphology, connectivity and physiology. Among these subtypes, those expressing vasoactive intestinal peptide (VIP), somatostatin (SST) and parvalbumin (PV) have important roles in regulating cortical circuits, with SST-INs mainly inhibiting excitatory neuron dendrites, PV-INs mainly inhibiting neuronal perisomatic regions, and VIP-INs mainly inhibiting other INs^11–15^. The precise regulation of the activity of these functionally diverse subtypes is essential for proper cortical circuit function and plasticity underlying healthy behaviour and learning. However, the effect of HD on distinct cortical IN subtypes is poorly explored, and direct evidence of the pathogenic interplay between cortical inhibition and CStr dysfunction is lacking.

Here we used longitudinal in vivo two-photon calcium imaging to examine the activity of VIP-INs, SST-INs and PV-INs, as well as of CStr neurons, in the motor cortex in HD model mice during spontaneous and learned movements throughout the disease progression. We uncovered a neuron subtype-specific dysfunction of cortical circuits in HD. We further demonstrate that subtype-specific activity modulation using optogenetics can normalize cortical network activity and provide a lasting improvement of motor deficits in HD mice.

## Cortical dysfunction in HD mice

**Fig. 1:**
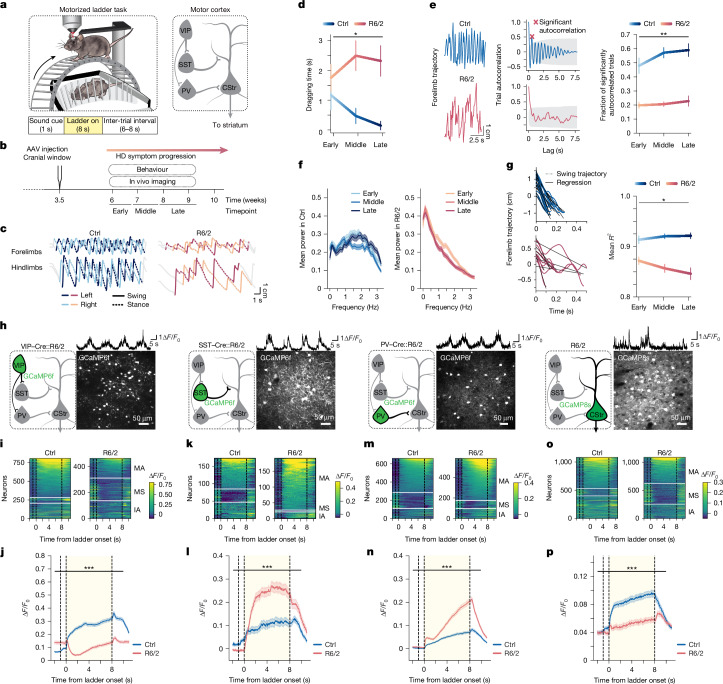
Motor deficits in HD mice accompany subtype-specific cortical neuron dysfunction. **a**, Schematic of the experimental setup and task structure (left), and a simplified connectivity scheme of the examined neuron types (right). **b**, Behavioural and imaging timeline across disease stages (Extended Data Fig. 1a; see Methods). **c**, Example forelimb and hindlimb trajectories in single trials in control (Ctrl) and R6/2 mice (middle timepoint). **d**, Hindlimb dragging duration per trial (aligned rank transform (ART); genotype *P* = 0.012; genotype × timepoint *P* = 0.017). **e**, Example trajectories (left), autocorrelation plots of example trials (middle) and the fraction of significantly autocorrelated trials (right; ART; genotype *P* < 0.001; genotype × timepoint *P* = 0.005). The grey shading indicates 95% confidence interval. **f**, Average power spectra of forelimb trajectories. **g**, Example swing trajectories with regression fits (left), and *R*^2^ values (right; ART; genotype *P* < 0.001; genotype × timepoint *P* = 0.037). *n* = 8 control and 8 R6/2 mice; 107 sessions total (**d**–**g**). **h**, Example two-photon images and calcium traces from VIP-INs (left), SST-INs (middle left), PV-INs (middle right) and CStr neurons (right) in M1. **i**, Trial-averaged VIP-IN activity sorted by movement modulation. Sample sizes (neurons/sessions/mice): *n* = 809/33/10 for Ctrl and 464/38/11 for R6/2. The dashed lines indicate cue, movement onset and offset (from left to right). IA, indiscriminately active; MA, movement active; MS, movement suppressed. **j**, Population average VIP-IN activity aligned to movement (ART; genotype *P* = 0.003; genotype × time *P* < 0.001). Yellow shading in **j**,**l**,**n**,**p** indicates the period of ladder rotation. **k**,**l**, SST-IN activity (neurons/sessions/mice: *n* = 173/15/5 for Ctrl; 191/19/4 for R6/2; ART; genotype *P* = 0.029; genotype × time *P* < 0.001). **m**,**n**, PV-IN activity (neurons/sessions/mice: *n* = 636/32/4 for Ctrl and 562/30/4 for R6/2; ART; genotype *P* = 0.088; genotype × time *P* < 0.001). **o**,**p**, CStr neuron activity (neurons/sessions/mice: *n* = 1,156/19/5 for Ctrl and 1,209/24/7 for R6/2; ART; genotype *P* = 0.031; genotype × time *P* < 0.001). Data are mean ± s.e.m. For this and all other figures: *P < 0.05, **P < 0.01 and ***P < 0.001 for interaction effects, and ^#^*P* < 0.05, ^##^*P* < 0.01 and ^###^*P* < 0.001 for group effects.

**Extended Data Fig. 1:**
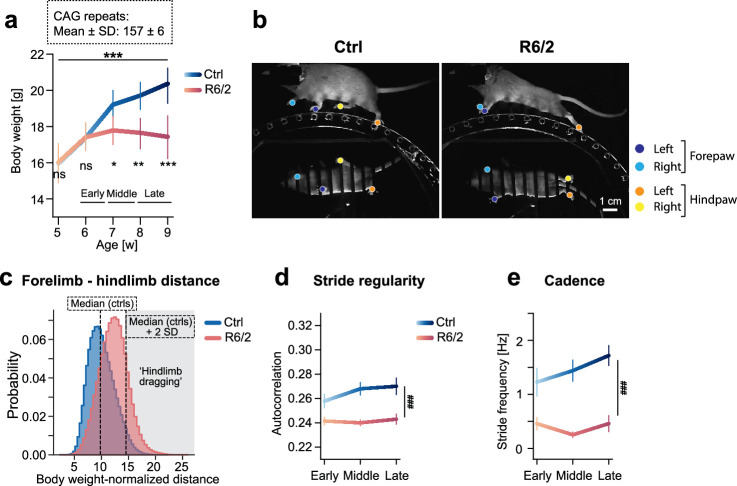
Motor deficits in R6/2 mice during the ladder task. (**a**) Body weights of the R6/2 mice used for the ladder task (157 ± 6 CAG repeats). R6/2 mice show normal weight at early stages, reduced weight gain at middle stages, and weight loss at late stages (n = 45 control and 61 R6/2 mice; ART; genotype × week p < 0.001; post hoc Mann–Whitney U tests per week: *p < 0.05, **p < 0.01, ***p < 0.001). (**b**). Example video frames of control and R6/2 mice at the middle stage. Fore- and hindpaw trajectories were extracted from side and bottom views. (**c**) Weight-normalized forepaw-hindpaw distance is longer in R6/2 mice due to hindlimb dragging, defined as the control median + 2 s.d. (n = 388 sessions from 33 control mice, 520 sessions from 44 R6/2 mice). (**d**) Autocorrelation of trial forelimb trajectories. Gait irregularity in R6/2 mice is reflected by a lower maximum autocorrelation (ART; genotype, *p* < 0.001). (**e**) Cadence is lower in R6/2 mice (ART; genotype, *p* < 0.001). (d-e) n = 8 control, 8 R6/2 mice; 107 sessions total. Data are mean ± s.e.m.

To investigate the neural mechanisms underlying motor symptoms in HD, we used the transgenic R6/2 mouse model of HD, which expresses exon 1 of the human mutant huntingtin (*mHTT*) gene and is characterized by motor impairments and reduced lifespan^16^. Non-transgenic littermates served as controls throughout. We trained mice on a motorized ladder task, in which mice learned to walk on a circular ladder with equally spaced rungs under head fixation in the dark. Following an auditory cue, the ladder rotated at a fixed speed for 8 s in each trial, during which mice had to walk on the ladder by grasping the rungs. The task requires precise forelimb placement, grasping and rapid adaptation—functions associated with the motor cortex. Mice were repeatedly tested in the task at early, middle and late learning or disease stages, which were defined by postnatal age corroborated by body weight and CAG repeat length (Fig. 1a,b and Extended Data Fig. 1a; see Methods). During the task, body movements were tracked by video analysis^17^ (Extended Data Fig. 1b). Compared with controls, R6/2 mice exhibited a range of deficits in movement kinematics (Fig. 1c), and we analysed a number of kinematic features to characterize these deficits. First, we quantified hindlimb dragging as a measure of failures to keep up with the rotation of the ladder (Extended Data Fig. 1c). R6/2 mice exhibited more hindlimb dragging than control mice, with the difference becoming more apparent over time (Fig. 1d). Next, we assessed the regularity of the gait pattern by an autocorrelation analysis of paw trajectories, which revealed a consistently lower regularity in the gait pattern of R6/2 mice (Fig. 1e and Extended Data Fig. 1d). We then analysed the distribution of cadence during gait, using Fourier analysis of paw trajectories. Although control mice showed a clear peak of cadence at approximately 1.3 Hz, indicating consistency of stride frequency, R6/2 mice lacked such a peak and showed overall reduced cadence (Fig. 1f and Extended Data Fig. 1e). In addition, the directness of strides—defined as the linearity of the forepaw trajectories during the swing phase of locomotion—was reduced in R6/2 mice and further deviated from control mice with disease progression (Fig. 1g). These various measures confirm motor deficits in R6/2 mice.

To link these behavioural abnormalities to cortical circuit function, we recorded the activity of several cortical neuron subtypes in mice during the ladder task with two-photon calcium imaging. We labelled VIP-INs, SST-INs and PV-INs by injecting AAV vectors expressing GCaMP6f in a Cre-dependent manner in the caudal forelimb area of the primary motor cortex (M1) of respective Cre lines^18^ (Fig. 1b,h). In R6/2 mice, VIP-INs exhibited a nearly complete absence of movement-related activity, including an abnormally large fraction of neurons suppressed during the movement period (Fig. 1i,j). By contrast, SST-INs and PV-INs were hyperactive during movements in R6/2 mice compared with the controls (Fig. 1k–n). To investigate how such subtype-specific dysregulations of cortical INs in R6/2 mice may be reflected in the activity of cortical principal neurons, we next examined CStr neurons projecting to the dorsolateral striatum, which is the part of the striatum most severely affected in HD. These CStr neurons were targeted by retrograde labelling to express GCaMP8s. We observed that CStr neuron activity during movement was significantly reduced in R6/2 mice compared with the controls (Fig. 1o,p).

**Extended Data Fig. 2:**
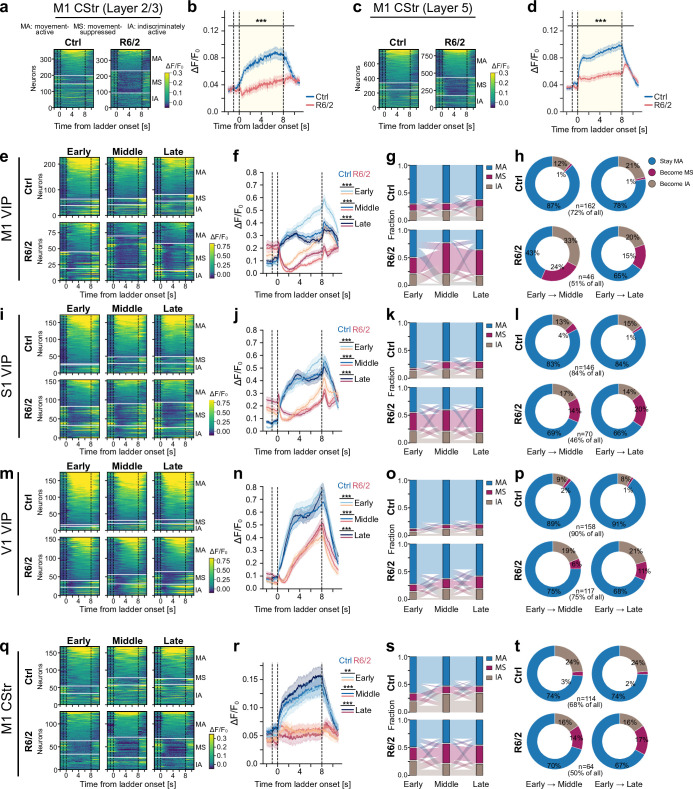
Circuit dysfunction is consistent across cortical layers, areas, and disease stages in the ladder task. (**a**,**b**) M1 L2/3 CStr neuron activity. (**a**) Trial-averaged single-neuron activity sorted by movement modulation; (**b**) population averages. Dashed lines indicate cue, movement onset, and offset. (ART; genotype p = 0.053; genotype × time bin p < 0.001). Sample sizes (neurons/sessions/mice): 359/6/3 control, 368/12/5R6/2 (depth 180–340 µm). (**c**,**d**) Same as (a,b) for M1 L5 CStr neurons (ART; genotype p = 0.188; genotype × time bin p < 0.001; sample sizes: 797/13/5 control, 841/12/5R6/2; depth 410–530 µm). (**e–h**) M1 VIP-INs tracked longitudinally across early, middle, and late stages. (**e**) Trial-averaged activity; (**f**) population averages (ART; genotype × time bin p < 0.001 for all stages). Sample sizes (neurons/sessions per timepoint/mice): 226/8/4 control, 91/7/3 R6/2. (**g**) transitions between movement-active (MA), movement-suppressed (MS), and indiscriminately active (IA) classes; (**h**) fraction of neurons remaining MA or changing class identity showing reduced stability in R6/2 mice. (**i-l**) Same as (e-h) for S1 VIP-INs (ART; genotype × time bin p < 0.001; sample sizes: 173/8/4 control, 153/8/4 R6/2). (**m-p**) Same as (e-h) for V1 VIP-INs (ART; genotype × time bin p < 0.001; sample sizes: 175/8/4 control, 157/8/4 R6/2). (**q-t**) Same as (e-h) for M1 CStr neurons (ART; genotype × time bin p = 0.007 (early), p < 0.001 (middle, late); sample sizes: 168/6/3 control, 127/6/3 R6/2). Mean ± s.e.m.

We characterized the anatomical specificity and temporal progression of the observed changes in more detail, focusing on VIP-INs and CStr neurons. CStr neurons in layer 2/3 (L2/3) and L5 of M1 both showed activity reductions (Extended Data Fig. 2a–d). VIP-IN hypoactivity was observed across M1, primary somatosensory (S1) and primary visual (V1) cortices. VIP-IN and CStr neuron activity was reduced across all timepoints of disease progression (Extended Data Fig. 2e,f,i,j,m,n,q,r). Tracking individual neurons across early, middle and late stages showed that, unlike controls, R6/2 mice exhibited progressive shifts away from movement-active classifications towards suppressed or inactive categories (Extended Data Fig. 2g,h,k,l,o,p,s,t), indicating a deterioration of normal functional responses over time.

**Extended Data Fig. 3:**
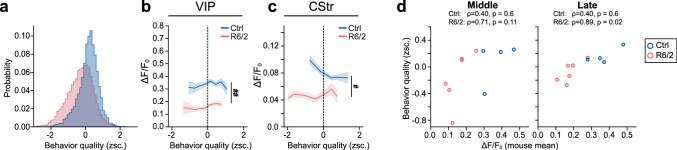
Cortical neuron dysfunction manifests across behavioural performance levels and is present in early stage zQ175DN HD mice. (**a**) Distribution of trial-by-trial composite behaviour scores (middle-late sessions, forelimb performance, z-scored across trials, see Methods) in control and R6/2 mice. Higher values indicate more coordinated gait (n = 261 sessions from 22 control mice, n = 291 sessions from 22 R6/2 mice). (**b,c**) Population activity as a function of behavioural performance. Trials were binned along the composite score axis (bin width = 0.3; ≥40 trials per genotype). (**b**) VIP-INs (ART; genotype p = 0.006; genotype × behaviour bin p = 0.060; sample sizes (trials/sessions/mice): 2,379/57/9 control, 2,471/59/8R6/2). (**c**) Same as (b) for CStr neurons (ART; genotype p = 0.044; genotype × behaviour bin p = 0.198; sample sizes: 1,714/37/8 control, 1,569 / 36 / 8 R6/2). Mean ± s.e.m. (**d**) Relationship between mean VIP-IN movement-related activity and behavioural score, shown for control and R6/2 mice at middle and late stages. Each dot represents one mouse (n = 4 control, 6 R6/2). Spearman correlations (ρ) and *p*-values are indicated.

Furthermore, VIP-INs and CStr neurons in R6/2 mice were hypoactive during movements across varying levels of movement quality (Extended Data Fig. 3a–c), demonstrating that the cortical activity abnormalities cannot be fully explained by altered movement kinematics, but instead reflect HD-specific dysfunction. Nevertheless, R6/2 mice with lower movement-related VIP-IN activity tended to have poorer behavioural performance, and this effect reached statistical significance at the late disease stage (Extended Data Fig. 3d). This relationship supports the interpretation that variability in VIP-IN recruitment contributes to animal-to-animal differences in behavioural outcome.

**Extended Data Fig. 4:**
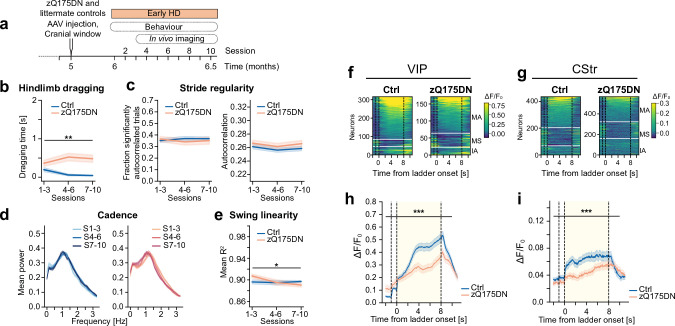
Cortical neuron-subtype-specific dysfunction in early-stage zQ175DN HD mice. (**a**) Behavioural and imaging schedule for zQ175DN experiments. (**b-e**) Behavioural performance in 6-month-old control and zQ175DN mice. (**b**) Hindlimb dragging duration (ART; genotype p = 0.001; genotype × session p = 0.022). (**c**) Gait regularity. Left, fraction of significantly autocorrelated trials (ART; genotype p = 0.733; genotype × session p = 0.264); right, autocorrelation (ART; genotype p = 0.496; genotype × session p = 0.980). (**d**) Trial-averaged power spectra of forelimb trajectories. (**e**) Stride linearity (R²; ART; genotype p = 0.752; genotype × session p = 0.015). (**f,g**) VIP-IN activity in zQ175DN mice. (f) Trial-averaged single-neuron activity; (g) population averages (ART; genotype p = 0.139; genotype × time bin p < 0.001; sample sizes (neurons/sessions/mice): 316/18/6 control, 171/12/4 zQ175DN). (**h,i**) Same as (f,g) for CStr neurons (ART; genotype p = 0.179; genotype × time bin p < 0.001; sample sizes: 434/17/6 control, 567/19/6 zQ175DN). Mean ± s.e.m.

To exclude the possibility that the observed dysfunctions are idiosyncratic to the R6/2 HD model, we also tested the knock-in zQ175DN mice, which express full-length mHTT and show slower disease progression than R6/2, at an early disease stage (6 months of age)^19,20^. zQ175DN mice showed mildly but significantly increased hindlimb dragging, but no overt impairment in other kinematic parameters (stride regularity, linearity and cadence) in the ladder task (Extended Data Fig. 4b–e). VIP-INs and CStr neurons were hypoactive during movement, although to a milder extent than in R6/2 mice (Extended Data Fig. 4f–i), indicating that the core cortical signature is consistent across HD models.

These results reveal neuron subtype-specific dysfunctions of the cortical circuit in HD. In particular, given the known connectivity among these neuron subtypes (VIP-INs inhibit SST-INs and PV-INs, which in turn inhibit CStr neurons; Fig. 1a), the absence of movement-related activity in VIP-INs could be partially responsible for the hyperactivity of PV-INs and SST-INs and hypoactivity of CStr neurons.

## IN dysfunction across behaviours

**Fig. 2:**
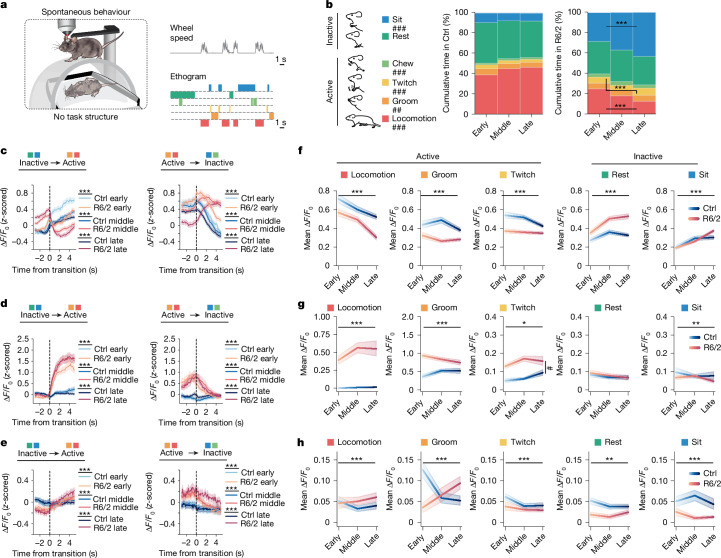
Abnormalities of spontaneous behaviours in HD mice accompany subtype-specific cortical IN dysfunction. **a**, Schematic of the experimental setup. Mice behaved on a passively rotating wheel without task structure. Calcium activity, wheel movement and the behaviour ethogram were recorded simultaneously. **b**, Cumulative duration of classified behavioural states (*n* = 26 Ctrl and 24 R6/2 mice; ART; genotype: locomotion *P* < 0.001, groom *P* = 0.008, twitch *P* < 0.001, chew *P* < 0.001, rest *P* = 0.463, sit *P* < 0.001; genotype × timepoint: locomotion *P* < 0.001, twitch *P* < 0.001, sit *P* < 0.001, others not significant (NS)). **c**–**e**, IN activity aligned to behavioural state transitions. Inactive → active (left) and active → inactive (right) transitions are shown, with their onset indicated by dashed lines. The average *z*-scored Δ*F*/*F*_0_ is shown for VIP-INs (**c**), SST-INs (**d**) and PV-INs (**e**) (ART, per timepoint; genotype × time *P* < 0.001 for all subtypes and timepoints). Sample sizes (neurons/sessions per timepoint/mice): VIP left: 883/33/8 for Ctrl and 817/33/9 for R6/2; VIP right: 788/29/8 for Ctrl and 780/32/9 for R6/2. SST left: 474/30/6 for Ctrl and 399/26/6 for R6/2; SST right: 442/29/6 for Ctrl and 296/20/6 for R6/2. PV left: 829/46/9 for Ctrl and 567/33/6 for R6/2; PV right: 628/36/9 for Ctrl and 532/32/6 for R6/2. **f**–**h**, Population activity during classified behaviours. VIP-IN activity was reduced (**f**), whereas SST-IN (**g**) and PV-IN (**h**) activities were increased during active states in R6/2 mice. ART; genotype × timepoint: for VIP, all behaviours *P* < 0.001; for SST, locomotion *P* < 0.001, groom *P* < 0.001, twitch *P* = 0.023 and others NS; and for PV, locomotion *P* < 0.001, groom *P* < 0.001, twitch *P* < 0.001, rest *P* = 0.003 and sit *P* < 0.001. Sample sizes (neurons/sessions per timepoint/mice): for VIP, 739–2,488/24–92/7–8 for Ctrl and 756–2,381/27–97/9 for R6/2; for SST, 207–1,152/10–62/4–6 for Ctrl and 344–946/19–54/6 for R6/2; and for PV, 556–1,663/25–77/8–9 for Ctrl and 273–653/13–31/5 for R6/2. Data are mean ± 95% confidence interval.

**Extended Data Fig. 5:**
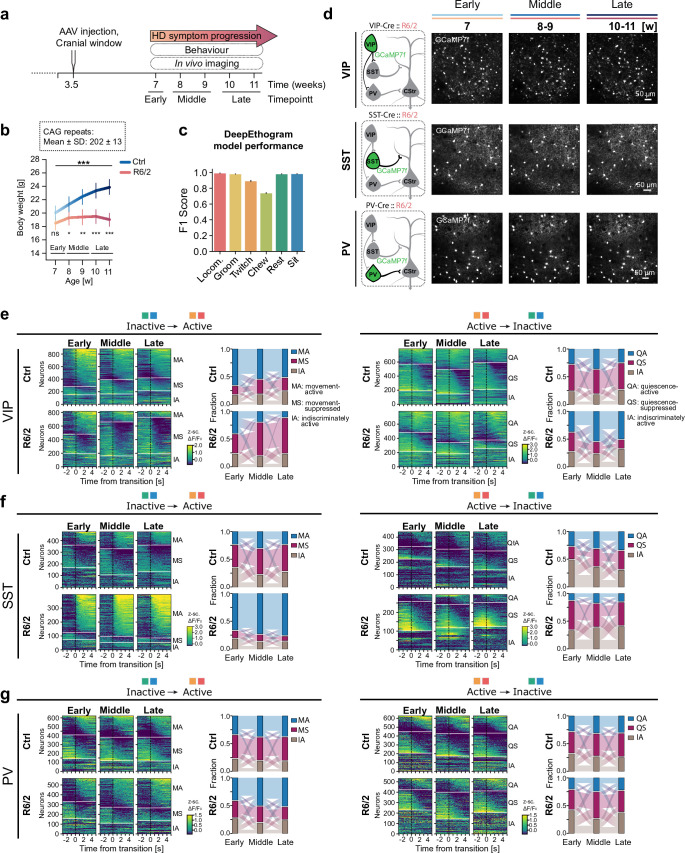
Additional analysis for the spontaneous behaviour paradigm on the wheel. (**a**) Experimental timeline for the wheel paradigm. (**b**) Body weights of R6/2 mice used in the wheel paradigm (202 ± 13 CAG repeats, ART; genotype × week, *p* < 0.001). (**c**) Classifier performance of DeepEthogram. F1 scores (precision–recall balance) are shown for each behavioural class for the final sequence model (see Methods). (**d**) Example maximum intensity projections of in vivo two-photon fluorescence images of VIP-, SST- and PV-INs. Identical fields of view were imaged at weekly intervals between 7 and 10–11 weeks of age, corresponding to early, middle, and late stages. (**e–g**) Interneuron activity across stages. z-scored ΔF/F_0_ from individual neurons aligned to behavioural state transitions (−3 s to +5 s). (**e**) VIP-INs, (**f**) SST-INs, (**g**) PV-INs. Sample sizes (neurons/sessions/mice; active/quiet): VIP, 883/788/32/29/8 control, 817/780/33/32/9 R6/2; SST, 474/442/30/29/6 control, 399/296/26/20/6 R6/2; PV, 829/628/46/36/9 control, 567/532/33/32/6 R6/2. Mean ± s.e.m.

To test whether the altered activity of cortical IN subtypes is generalizable across different movements, we used another behavioural paradigm in which head-fixed mice were placed on a continuous-surface wheel that mice could turn by spontaneous locomotion. This was done repeatedly for early, middle and late phases as described above (Extended Data Fig. 5a,b). We did not impose any task structure and instead observed the spontaneous behaviour of the mice examined by video analysis (Fig. 2a). Using a semi-supervised deep learning algorithm^21^ applied on videos, behavioural epochs were classified into different behavioural categories (Fig. 2a and Extended Data Fig. 5c). These categories of spontaneous behaviours included ‘active’ epochs such as ‘locomotion’, ‘grooming’ and forelimb ‘twitches’, as well as ‘inactive’ epochs corresponding to periods labelled as ‘rest’ and ‘sit’ (Methods). Control mice showed a distribution of these epochs that were consistent throughout the timepoints, whereas R6/2 mice demonstrated a different distribution at the early stage, as well as a progressive deviation from the pattern seen in the control mice. Specifically, the cumulative duration of active epochs significantly decreased and inactive epochs correspondingly increased (Fig. 2b). The decrease of active epochs in R6/2 mice was largely accounted for by a decrease in locomotion periods.

**Extended Data Fig. 6:**
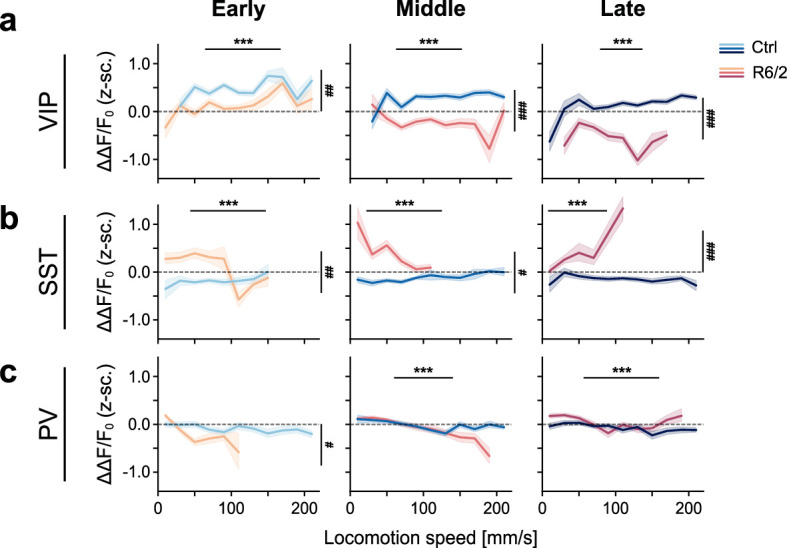
Cortical neuron dysfunction is independent of locomotion speed. (**a**) Changes in z-scored calcium activity (ΔΔF/F_0_; −3 s to +5 s around locomotion onset) as a function of locomotion speed, shown for early, middle, and late stages. Δspeed values were binned (width = 20 mm/s; sample sizes (bouts/sessions/mice): 44,160/157/8 control, 13,363/122/9 R6/2); ART; Early: genotype, *p* = 0.005; genotype × speed bin, *p* < 0.001; Middle: genotype, *p* < 0.001; genotype × speed bin, *p* < 0.001; Late: genotype, *p* < 0.001; genotype × speed bin, *p* < 0.001). (**b**) Same as (a), but for SST-INs (n = 16,321/123/6 control, 6,204/83/6 R6/2; ART; Early: genotype, *p* = 0.004; genotype × speed bin, *p* < 0.001; Middle: genotype, *p* = 0.020; genotype × speed bin, *p* < 0.001; Late: genotype, *p* < 0.001; genotype × speed bin, *p* < 0.001). (**c**) Same as (a), but for PV-INs (50,813/238/10 control, 34,025/172/7 R6/2; ART; Early: genotype, *p* = 0.027; genotype × speed bin, *p* = 0.050; Middle: genotype, *p* = 0.144; genotype × speed bin, *p* < 0.001; Late: genotype, *p* = 0.210; genotype × speed bin, *p* < 0.001). Mean ± c.i.

We proceeded to evaluate cortical IN activity during this behavioural paradigm in R6/2 and control mice. To achieve this, we expressed Cre-dependent GCaMP7f in M1 of VIP–Cre, SST–Cre and PV–Cre lines^22^, crossed to R6/2 mice and monitored VIP-IN, SST-IN and PV-IN activity longitudinally over 4–5 weeks, spanning early, middle and late disease stages (Extended Data Fig. 5d). R6/2 mice displayed abnormal IN activity. In control mice, VIP-IN activity was higher during active behavioural states than inactive states. However, this modulation was reversed in R6/2 mice, in which VIP-INs became less active at the onset of active motor behaviours and more active at transitions to inactive behavioural states (Fig. 2c and Extended Data Fig. 5e). In contrast to VIP-INs, SST-INs in control mice showed only moderate activity during active behaviours, whereas SST-INs in R6/2 mice exhibited much more pronounced movement-related activity (Fig. 2d and Extended Data Fig. 5f). In addition, PV-IN activity was elevated during movement in R6/2 mice (Fig. 2e,h and Extended Data Fig. 5g). These patterns were consistent across disease progression and evident at the level of single-neuron distributions (Extended Data Fig. 5e–g). Hypoactivity of VIP-INs and hyperactivity of SST-INs during locomotion were observed regardless of the locomotion speed in each bout, indicating that the dysfunctions are not due to differences in movements (Extended Data Fig. 6a,b).

We evaluated IN activity alterations during episodes of all classified behaviours. In R6/2 mice, we observed a marked reduction in VIP-IN activity, and an increase in SST-IN and a partial increase in PV-IN activity during movements (locomotion, grooming and twitching), changes that were either absent or reversed during inactive states (rest and sit) (Fig. 2f–h).

**Extended Data Fig. 7:**
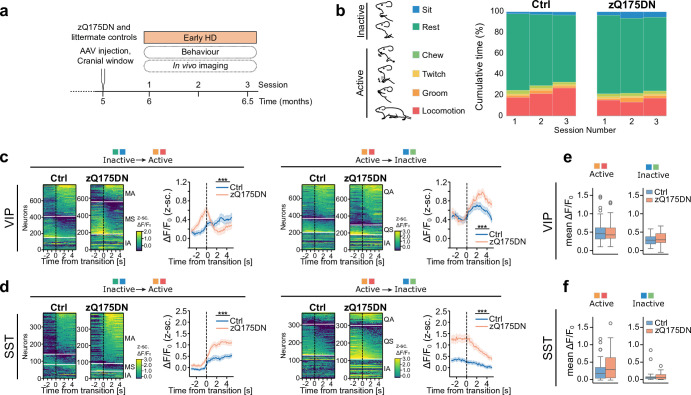
Cortical neuron-subtype alteration in early-stage zQ175DN mice during spontaneous behaviours. (**a**) Behavioural and imaging schedule. (**b**) Cumulative duration of classified behavioural states. (n = 64 sessions from 8 control mice; n = 44 sessions from 7 zQ175DN mice; Aligned rank transform; n.s. for all comparisons) (**c**) Abnormal VIP-IN modulation in zQ175DN mice at transitions from inactive to active behavioural states and vice versa. Average z-scored ΔF/F_0_ aligned to state transitions (ART; genotype × time bin, *p* < 0.001; sample sizes (neurons/sessions/mice): left, 797/24/4 control, 756/27/4 zQ175DN; right, 761/23/4 control, 771/27/4 zQ175DN). (**d**) Same as (c), but for SST-INs (ART; genotype × time bin, *p* < 0.001; sample sizes: left, 380/33/4 control, 397/24/3 zQ175DN; right, 367/32/4 control, 369/21/3 zQ175DN). Mean ± c.i. (**e**) Average VIP-IN population activity during active (left) or inactive (right) behaviour bouts is not significantly different between control and zQ175DN mice. (**f**) Same as (e), but for SST-INs.

We again sought to confirm these results in early symptomatic, 6-month-old zQ175DN mice (Extended Data Fig. 7b). The distribution of spontaneous active and inactive behaviours on the wheel were comparable across genotypes (Extended Data Fig. 7b). We observed VIP-IN hypoactivity and SST-IN hyperactivity particularly at the onset of spontaneous active behaviours, similarly to R6/2 mice albeit with milder effect sizes (Extended Data Fig. 7c–f). This indicates that these cortical IN alterations are consistent across HD models.

Together, these results reveal that, similarly to skilled locomotion in the ladder task, spontaneous movements in HD mice are accompanied by a consistent alteration in the activity of cortical IN subtypes. VIP-INs demonstrated hypoactivity and SST-INs and PV-INs showed hyperactivity during movements, providing evidence for increased inhibition onto excitatory neurons during active movement states.

## Persistent rescue of cortical activity

The neuron subtype-specific alteration of activity in HD affords a potential opportunity for intervention to normalize cortical network function by a targeted modulation of genetically defined neuron subtypes. VIP-INs are upstream of the other observed neuron types^11–13,23^, and their hypoactivity in R6/2 mice could disinhibit other INs leading to their hyperactivity, which would in turn result in increased inhibition of CStr neurons explaining their hypoactivity. We hypothesized that artificial stimulation of VIP-INs could correct the hypoactivity of CStr neurons in R6/2 mice. Therefore, we chose VIP-INs as the target for manipulation using optogenetics.

Neural activity modulation in diseases has an enormous therapeutic potential but may require a careful selection of the artificial modulation parameters to achieve physiological levels of neural activity^24^. Taking this into account, we evaluated the effect of our VIP-IN stimulation protocol on VIP-IN activity associated with movement.

**Fig. 3:**
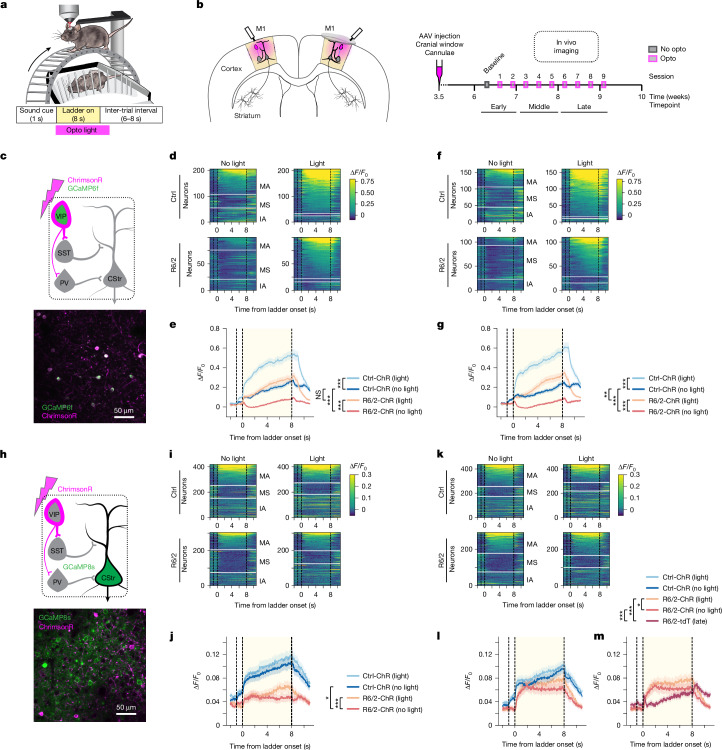
Optogenetic activation of VIP-INs normalizes VIP-IN and CStr neuron activity. **a**, Schematic of VIP-IN stimulation and imaging during the ladder task. **b**, Experimental timeline. Bilateral ChR expression in M1 VIP-INs with the cranial window over the right hemisphere and bilateral light-dispersing cannulas (left). After one baseline session, mice were trained with stimulation in 40% of trials every other day; imaging was performed at the middle stage (sessions 4 and 5) and late stage (sessions 7 and 8) (right). **c**, Example two-photon image showing ChR and GCaMP6f co-expression in VIP-INs. **d**,**e**, VIP-IN activity at the middle stage, presented as single-neuron activity (**d**) and trial-averaged responses (**e**; light is denoted by lighter hues, and no light is indicated by darker hues). Dashed lines in **d**–**g** and **i**–**m** indicate cue, movement onset and offset (from left to right). Yellow shading in **e**,**g**,**j**,**l**,**m** indicates the period of ladder rotation. ART: no light genotype *P* = 0.006, genotype × time *P* < 0.001, trial type (R6/2) *P* < 0.001, trial type × time *P* < 0.001; ctrl no light versus R6/2 light, genotype *P* = 0.533, genotype × time *P* = 0.415. Sample sizes (neurons/sessions/mice): 206/5/3 for Ctrl and 100/4/3 for R6/2. **f**,**g**, Same as in **d**,**e** but for the late stage. ART: no light genotype *P* = 0.025, genotype × time *P* < 0.001, trial type (R6/2) *P* < 0.001, trial type × time *P* < 0.001; ctrl no light versus R6/2 light, genotype *P* = 0.616, genotype × time *P* = 0.002. Sample sizes (neurons/sessions/mice): 160/5/3 for Ctrl and 110/4/3 for R6/2. **h**, Example two-photon image showing ChR expression in VIP-INs and GCaMP8s in CStr neurons. **i**,**j**, CStr neuron activity at the middle stage, presented as single-neuron activity (**i**) and population averages (**j**). ART: no light genotype *P* = 0.033, genotype × time *P* = 0.024, trial type (R6/2) *P* < 0.001, trial type × time *P* < 0.001; ctrl no light versus R6/2 light, genotype *P* = 0.038, genotype × time *P* = 0.938. Sample sizes (neurons/sessions/mice): 418/6/3 for Ctrl and 303/8/3 for R6/2. **k**,**l**, Same as in **i**,**j** but for the late stage. ART: no light genotype *P* = 0.024, genotype × time *P* = 0.306, trial type (R6/2) *P* < 0.001, trial type × time *P* = 0.018; ctrl no light versus R6/2 light, genotype *P* = 0.153, genotype × time *P* = 0.566. Sample sizes (neurons/sessions/mice): 437/6/3 for Ctrl and 292/8/3 for R6/2. **m**, Comparison of all trials in R6/2-tdT mice versus no light and light trials in R6/2-ChR mice, replotted from **j** and Extended Data Fig. 8j. ART: actuator main effects NS; actuator × time *P* < 0.001 for both light and no light conditions. Data are mean ± s.e.m.

**Extended Data Fig. 8:**
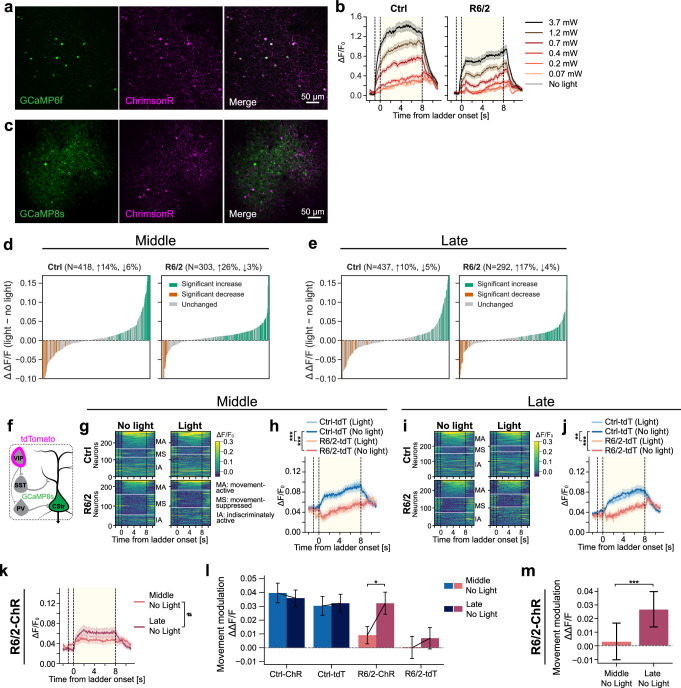
Optogenetic activation of VIP-INs normalizes VIP-IN and CStr neuron activity in HD mice. (**a**) Maximum intensity projections of two-photon images shown as cropped overlay images in Fig. 3c, detailing ChrimsonR and GCaMP6f co-expression in VIP-INs. (**b**) Light intensity–dependent activation of VIP-INs in control and R6/2 mice. Pairs of light and no-light trials alternated, with increasing light power every 8 trials (sample sizes (neurons/sessions / mice): 184/4/2 control, 137/4/2 R6/2). (**c**) Maximum intensity projections of two-photon images shown as cropped overlay images in Fig. 3h, detailing ChrimsonR expression in VIP-INs and GCaMP8s expression in CStr neurons. (**d**,**e**) Changes in movement-related activity (ΔΔF/F light − no light) for individual CStr neurons at middle (d) and late (e) stages. Each bar represents one neuron. Cells with significant increases (teal) or decreases (orange) were identified by Welch’s t-test with false discovery rate correction (q < 0.05). (**f**) Schematic of no-opsin control experiments with tdTomato expression in VIP-INs and CStr imaging. (**g**,**h**) CStr activity in no-opsin controls at the middle stage. (g) Trial-averaged activity; (h) population averages (sample sizes: 267/5/4 control, 232/8/5 R6/2; ART; genotype × time bin p < 0.001 for both trial types). (**i**,**j**): Same as (g,h), but for the late stage (ART; genotype × time bin p < 0.01 for both trial types). No differences were observed between light and no-light trials in either genotype. (**k**) Trial-averaged CStr activity in R6/2–ChR mice during middle vs late sessions in no-light trials, re-plotted from Fig. 3j,l. ART; timepoint, *p* = 0.022. (**l**) Population-level movement modulation (ΔF/F_0_ during movement minus baseline) across Ctrl-ChR, Ctrl-tdT, R6/2-ChR and R6/2–ChR mice. Linear mixed models with mice as random intercepts; group × timepoint p = 0.040; R6/2-ChR timepoint effect p = 0.019; others n.s. (**m**) Within-neuron changes in movement modulation for longitudinally tracked CStr neurons in R6/2-ChR mice (sample sizes: 93/3/2 neurons/sessions/mice per timepoint; linear mixed model with mouse and cell random intercepts, p < 0.001). Mean ± s.e.m.

We co-expressed the excitatory opsin ChrimsonR (ChR) and GCaMP6f in VIP-INs in M1 of R6/2 mice to simultaneously activate these neurons and image their activity (Fig. 3a,c and Extended Data Fig. 8a). VIP-INs showed stronger activation with higher light intensities (Extended Data Fig. 8b). In particular, 0.7-mW stimulation increased VIP-IN activity in R6/2 mice to the level comparable with the controls, whereas stronger light intensity drove VIP-IN activity to levels higher than the controls without stimulation.

We next examined both acute and persistent effects of 0.7-mW VIP-IN stimulation on VIP-IN and CStr activity. At 6 weeks (early stage), mice performed a baseline ladder session without stimulation. They then completed nine additional sessions every other day covering early, middle and late timepoints, with randomly interleaved 40% of trials with optogenetic stimulation. Imaging was performed in the middle phase (sessions 4 and 5) and late phase (sessions 7 and 8) (Fig. 3b). During no-light trials, VIP-INs in R6/2 mice remained hypoactive compared with controls in both middle and late stages (Fig. 3d–g), consistent with the previous experiment (Fig. 1i,j and Extended Data Fig. 2e–h). Light trials produced robust activation, elevating R6/2 VIP-IN activity to the range of control activity for no-light trials at both the middle disease stage (Fig. 3d,e) and the late disease stage (Fig. 3f,g). These results indicate that our stimulation can effectively restore VIP-IN activity throughout disease progression in a light-dependent manner.

**Extended Data Fig. 9:**
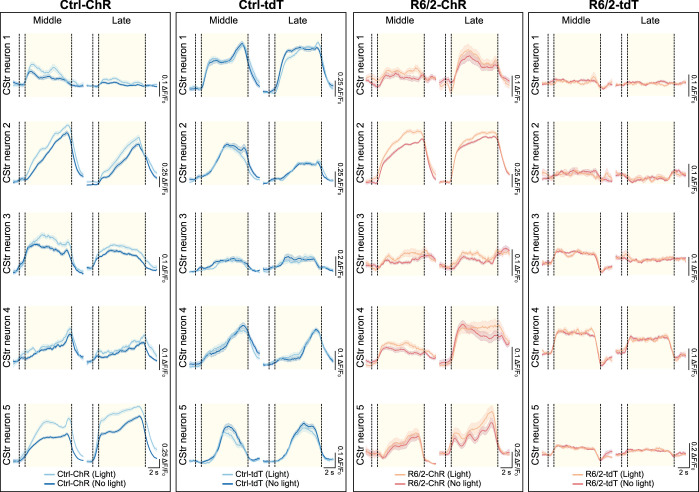
Examples of the activity of longitudinally tracked single CStr neurons. Trial-averaged activity of example CStr neurons in middle (sessions 4 and 5) and late (sessions 7 and 8) stages, from 2 s before ladder onset to 3 s after ladder offset, shown separately for light and no-light trials. Dashed lines indicate cue, ladder onset, and ladder offset. Five individual neurons from four experimental groups each (Ctrl-ChR, Ctrl, tdT, R6/2-ChR and R6/2-tdT) are shown as indicated. Mean ± s.e.m.

To assess downstream consequences, we imaged CStr neurons while stimulating VIP-INs under the same conditions (Fig. 3h–m). At the middle stage, as expected, CStr activity was lower in no-light trials of R6/2 mice than in control mice (Fig. 3i,j). VIP-IN activation produced an acute, modest increase in CStr activity in R6/2 mice (light versus no-light trials), consistent with disinhibition (Fig. 3i and Extended Data Fig. 9). Nevertheless, this acute effect did not fully restore CStr activity to the control levels at the middle stage (Fig. 3i,j and Extended Data Fig. 8d,e). Light delivery alone had no effect in the absence of ChR (Extended Data Fig. 8f–j). By the late stage, we observed an accumulated benefit: in stimulated R6/2 mice, CStr activity in no-light trials was higher than in the middle stage (Extended Data Fig. 8k,l). This middle-to-late stage improvement was also observed in the subset of the data in which the same neurons were longitudinally tracked (Extended Data Figs. 8m and 9). Accordingly, in the no-light trials at the late stage, CStr neuron activity of the stimulated R6/2 mice was higher than in non-stimulated R6/2 mice, at the level similar to no-light trials in control animals (Fig. 3k–m). Together, these results support a model in which repeated VIP-IN activation drives cumulative circuit reorganization that outlasts acute stimulation, presumably by enhancing synaptic plasticity.

These observations demonstrate that targeted VIP-IN activation can restore activity in downstream CStr neurons and underscore the potential of circuit-level interventions to ameliorate network deficits in HD.

## VIP-IN activation ameliorates motor symptoms

**Fig. 4:**
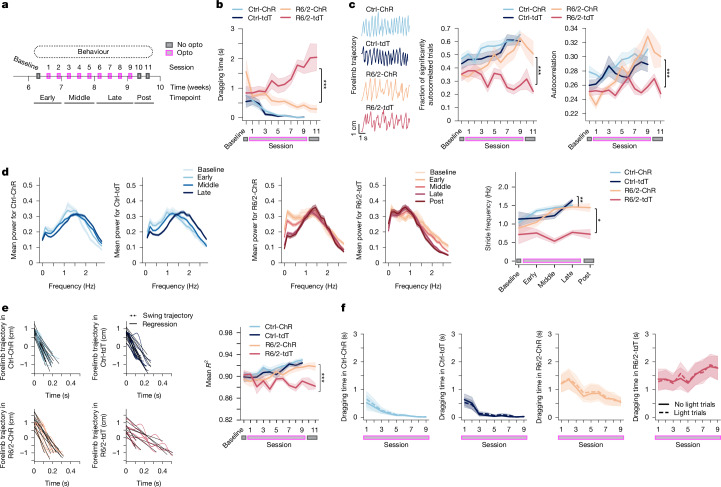
VIP-IN activation improves motor learning and provides lasting behavioural benefits in HD mice. **a**, Experimental timeline. After one baseline session, mice were trained with optogenetic stimulation in 40% of trials every other day for nine sessions, followed by two post-stimulation sessions without light. Control data were pooled across 0.7-mW and 3.7-mW experiments, owing to limited controls in the 0.7-mW experiment and no power effect in controls; R6/2 data are from the 0.7-mW cohort. **b**, Hindlimb dragging duration per trial decreased in Ctrl-ChR, Ctrl-tdT and R6/2-ChR mice (ART; actuator × session, *P* = 0.090 for Ctrl and *P* < 0.001 for R6/2). Optogenetic improvement of R6/2 behaviour persisted in the sessions after stimulation (sessions 10 and 11). **c**, Example forelimb trajectories (late stage; left), fraction of significantly autocorrelated trials (middle), and maximum autocorrelation (right). Gait regularity increased with learning in Ctrl-ChR, Ctrl-tdT and R6/2-ChR mice (ART; actuator × session: for fraction, *P* = 0.445 for Ctrl and *P* < 0.001 for R6/2; for autocorrelation, *P* = 0.237 for Ctrl and *P* < 0.001 for R6/2). **d**, Trial-averaged power spectra (left); R6/2-ChR spectra shift towards resembling control spectra (middle); and mean peak frequencies of the plots on the left (right). Cadence increased over sessions (ART; actuator × session: *P* = 0.003 for Ctrl and *P* = 0.012 for R6/2). **e**, Example stride trajectories with regression fits (left), and mean *R*^2^. Stride linearity increased with learning in Ctrl and R6/2-ChR mice (right; ART; actuator × session: *P* = 0.787 for Ctrl and *P* < 0.001 for R6/2). Sample sizes (mice): *n* = 15 Ctrl-ChR, 11 Ctrl-tdT, 7 R6/2-ChR and 6 R6/2-tdT; 407 sessions total (**b**–**e**). **f**, Persistence of behavioural effects beyond stimulation. Hindlimb dragging compared between light and no-light trials is shown (ART; trial type × session interaction: *P* = 0.638 for Ctrl-ChR, *P* = 0.982 for Ctrl-tdT, *P* = 0.995 for R6/2-ChR and *P* = 0.997 for R6/2-tdT). Sample sizes (mice): *n* = 15 Ctrl-ChR, 11 Ctrl-tdT, 18 R6/2-ChR and 17 R6/2-tdT; 542 sessions total. Data are mean ± s.e.m.

**Extended Data Fig. 10:**
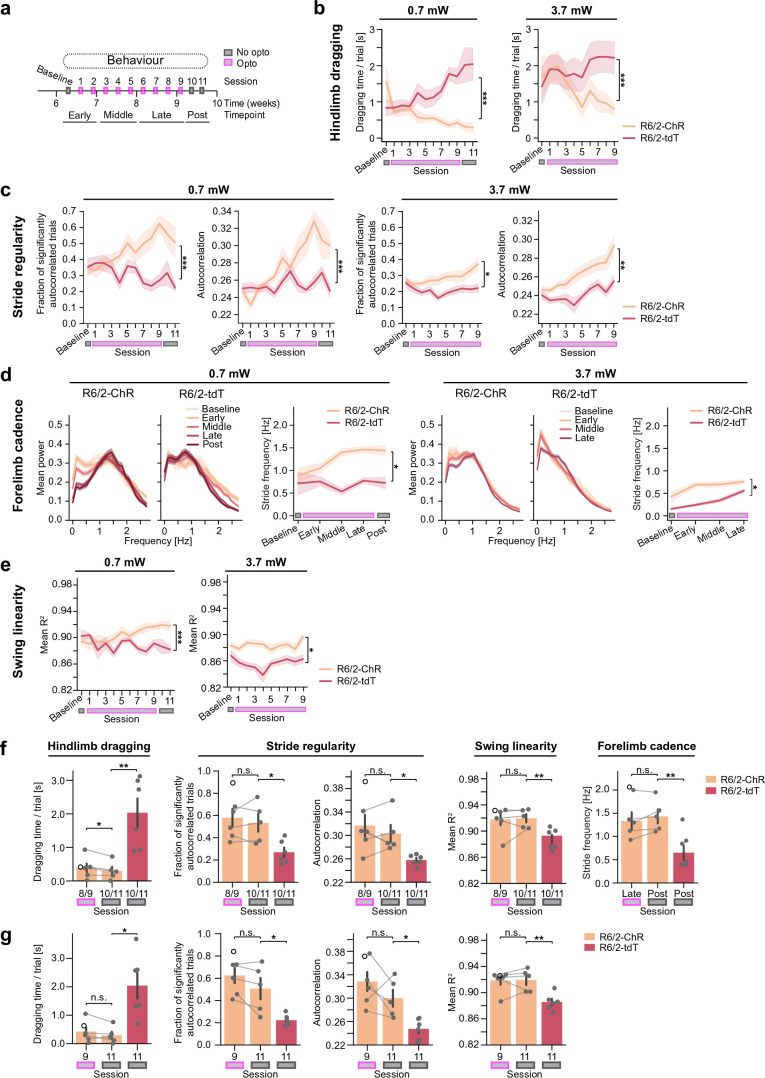
Behavioural benefits of VIP-IN stimulation across stimulation intensities. (**a**) Experimental timeline. After one baseline session, mice were trained with optogenetic stimulation in 40% of trials every other day for 9 sessions, followed by two post-stimulation sessions without light. (**b–e**) Behavioural performance in R6/2 mice at two light intensities (0.7 mW and 3.7 mW). (**b**) Hindlimb dragging duration decreased with stimulation (ART; actuator × session p < 0.001 for both powers). (**c**) Gait regularity. Fraction of significantly autocorrelated trials and maximum autocorrelation increased over sessions in R6/2-ChR mice (ART; actuator × session: fraction, p < 0.001 (0.7 mW), p = 0.044 (3.7 mW); autocorrelation, p < 0.001 (0.7 mW), p = 0.018 (3.7 mW)). (**d**) Cadence increased with training and stimulation in R6/2-ChR mice (ART; actuator × session: p = 0.012 (0.7 mW), p = 0.015 (3.7 mW)). (**e**) Stride linearity (R²) increased with stimulation (ART; actuator × session: p < 0.001 (0.7 mW), p = 0.020 (3.7 mW); sample sizes (sessions): 261 (0.7 mW), 412 (3.7 mW)). (**f**) Persistence of behavioural effects after stimulation. Comparisons between late stimulation sessions (8–9) and post sessions (10–11) in R6/2-ChR mice were performed using linear mixed models (mouse as random intercept): dragging time p = 0.015; fraction p = 0.750; autocorrelation p = 0.978; cadence p = 0.074; R² p = 0.504. Between-group comparisons (R6/2-ChR vs R6/2-tdT, post sessions; Welch’s t-test): dragging time p = 0.008; fraction p = 0.025; autocorrelation p = 0.037; cadence p = 0.010; R² p = 0.006. (**g**) Same as (f) with comparisons for session 9 vs session 11. Within-group (Wilcoxon signed-rank): dragging time p = 0.312; fraction p = 0.125; autocorrelation p = 0.312; R² p = 0.625. Between-group (Welch’s t-test): dragging time p = 0.010; fraction p = 0.040; autocorrelation p = 0.018; R² p = 0.007. Mean ± s.e.m.

**Extended Data Fig. 11:**
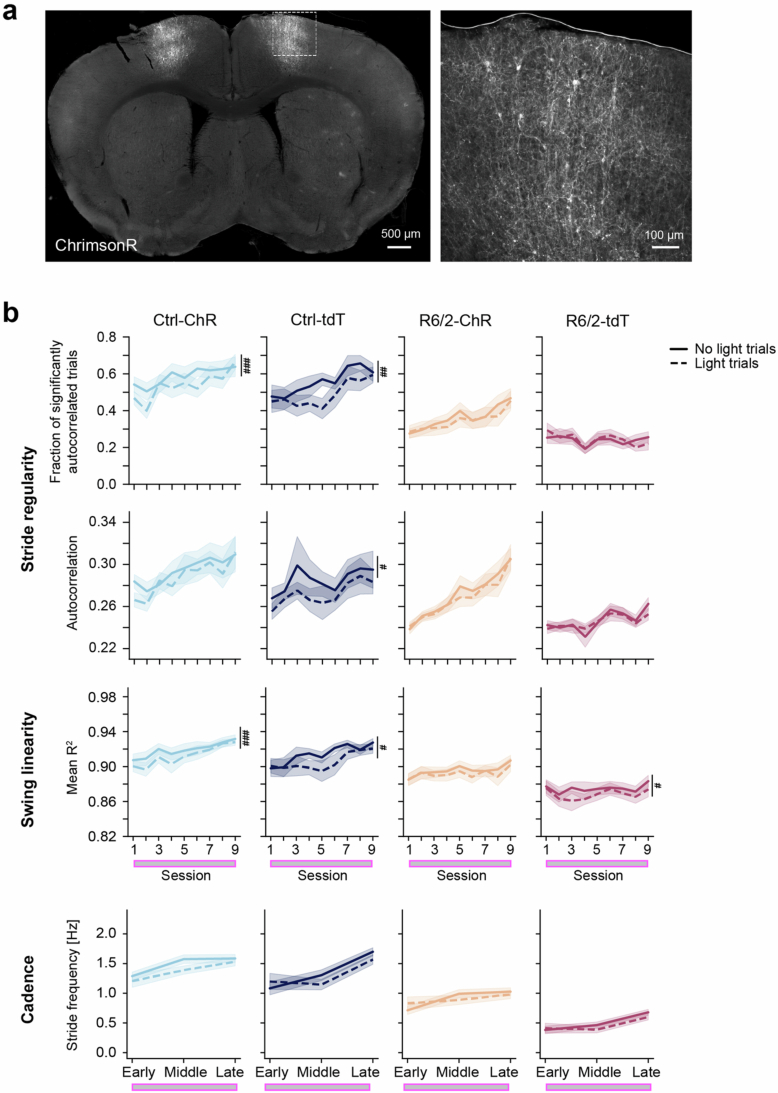
Additional behavioural data from bilateral VIP-IN activation. (**a**) Example coronal section from an R6/2 mouse expressing ChrimsonR in VIP-INs in M1. Left: whole section. Right: magnified image from the motor cortex, area indicated by the boxed region on the left. (**b**) Comparison of light and no-light trials across behavioural metrics. ART; trial type main effects are shown for each group. Stride regularity (fraction of significantly autocorrelated trials): Ctrl-ChR p < 0.001, Ctrl-tdT p < 0.001, R6/2-ChR p = 0.115, R6/2-tdT p = 0.674. Autocorrelation: Ctrl-ChR p = 0.318, Ctrl-tdT p = 0.016, R6/2-ChR p = 0.120, R6/2-tdT p = 0.317. Stride linearity (R²): Ctrl-ChR p < 0.001, Ctrl-tdT p = 0.013, R6/2-ChR p = 0.100, R6/2-tdT p = 0.020. Cadence: Ctrl-ChR p = 0.095, Ctrl-tdT p = 0.466, R6/2-ChR p = 0.882, R6/2-tdT p = 0.087. Post hoc Mann–Whitney *U-* tests showed no significant differences across individual sessions (see Supplementary Table 1). Sample sizes (mice/sessions): 15 Ctrl-ChR, 11 Ctrl-tdT, 18 R6/2-ChR, 17 R6/2-tdT; 542 sessions total. Data include both 0.7 mW and 3.7 mW stimulation conditions. Data are mean ± s.e.m.

We next explored whether the restoration of cortical network activity improves motor behaviour in R6/2 mice. To address this, we used the same stimulation protocol to repeatedly activate VIP-INs bilaterally and examined its effects on motor performance in R6/2 mice (Fig. 4a and Extended Data Figs. 10a and 11a; see Methods). We tested two optogenetic stimulation regimes: a regime identical to the imaging experiments (0.7 mW) to restore healthy VIP-IN activity and a stronger stimulation (3.7 mW) that activates VIP-INs beyond what is observed in healthy mice.

Across sessions, control mice demonstrated motor learning and improved their task performance, evidenced by reduced hindlimb dragging, increased autocorrelation of gait trajectories, a clearer peak in cadence rhythm and increased regularity of strides (Fig. 4b–f and Extended Data Fig. 11b). Consistent with the previous results (Fig. 1d–g), R6/2 mice expressing tdTomato (R6/2-tdT) as a control showed persistent hindlimb dragging without improvement, irregular gait patterns without a coherent cadence rhythm and poor coordination of strides (Fig. 4b–f and Extended Data Figs. 10b–e and 11b). By contrast, R6/2 mice that received VIP-IN stimulation displayed a progressive decrease in hindlimb dragging over time (Fig. 4b and Extended Data Fig. 10b), and more regular stride patterns than R6/2-tdT mice (Fig. 4c and Extended Data Fig. 10c). Furthermore, R6/2 mice receiving VIP-IN stimulation demonstrated cadence changes similar to control mice, adopting faster and more consistent strides over time (Fig. 4d and Extended Data Fig. 10d), as well as substantial improvements in the directness of stride swings (Fig. 4e and Extended Data Fig. 10e). The behavioural improvements were observed in both stimulation strengths (0.7 mW and 3.7 mW; Extended Data Fig. 10), indicating that behavioural rescue is robust across stimulation parameters.

In the analysis above, the light trials (40%) and no-light trials (60%) were pooled together. We next sought to examine whether the beneficial effects of VIP-IN stimulation were only during the stimulation, or whether they persisted beyond stimulation. To this goal, we compared the behavioural performance in light and no-light trials. We did not find any difference in behavioural performance between these trial types in R6/2 mice with VIP-IN stimulation (Fig. 4f and Extended Data Fig. 11b), indicating that the behavioural improvements persisted beyond the stimulation periods. To test whether this effect lasts across days, we performed two additional behavioural sessions after the last stimulation in the 0.7-mW regime (‘post’ sessions 10 and 11, corresponding to 2 and 4 days after the last stimulation, respectively). Stimulated R6/2 mice maintained their improved performance in these post-stimulation sessions, displaying significantly better performance than that of non-stimulated R6/2 mice (Fig. 4a–e and Extended Data Fig. 10b–g). Within the stimulated R6/2 cohort, performance in the post-stimulation sessions was comparable with the last stimulation sessions across all four behavioural measures (Extended Data Fig. 10f,g). Together, these results indicate that VIP-IN stimulation can mitigate motor deficits in HD mice and that these benefits persist for days after treatment cessation.

## Discussion

The symptoms of neurological disorders arise from a complex interplay between genetic, molecular and circuit-level dysfunctions, complicating rational therapeutic design, even for monogenic disorders with a clearly defined genetic origin, such as HD. Identifying the critical nodes of the complex dysfunctions would illuminate intervention strategies, as modulation of these nodes could correct multiple downstream processes.

Cortical dysfunction in HD is among the earliest pathological signatures, which includes an imbalance between excitation and inhibition^25–27^. Studies in conditional HD mouse models have found that mHTT expression in cortical INs and principal neurons both contribute to the development of full-fledged cortical HD pathology and behavioural defects^10,28^. Furthermore, CStr neurons in the motor cortex appear selectively vulnerable in HD^29^. Although previous work suggests differential contributions of cortical neuron subtypes to driving behavioural symptoms^26,30,31^, the roles of specific inhibitory and excitatory neuron populations remain poorly understood.

Here we examined the activity patterns of three cortical IN subtypes and CStr neurons in the well-established R6/2 HD mouse model^16^ in vivo. Across identified behaviours, we observed a consistent cortical signature: VIP-IN hypoactivity together with SST-IN and PV-IN hyperactivity and reduced CStr neuron output. We recognize that R6/2 is a rapid, early-onset model that does not capture all features of adult-onset HD in humans. We addressed this limitation by demonstrating similar VIP-IN, SST-IN and CStr neuron alterations in zQ175DN mice in both behavioural paradigms with milder effect sizes, indicating that cortical abnormalities generalize across models.

Among these abnormalities, VIP-IN hypoactivity emerged as an upstream leverage point. Selective optogenetic activation of VIP-INs in R6/2 mice acutely increased CStr neuron activity and, with repeated stimulation, led to a sustained improvement in CStr activity levels. This intervention within the motor cortex improved motor deficits over time. VIP-INs are a unique group of cortical INs with preferential connections onto other INs. Thus, their activation can reduce both dendritic (SST-IN) and perisomatic (PV-IN) inhibition of principal cells and thereby disinhibit excitatory output. It is striking that the artificial pattern of stimulation benefited behaviour across stimulation strengths, given that behaviour is controlled by precise and complex spatiotemporal patterns of neural activity. We speculate that our manipulation of inhibitory circuits effectively reduced the pathologically strong levels of inhibition on excitatory neurons, such that excitatory neurons could now function more normally to express their healthy activity patterns. We note that HD involves widespread cortical and subcortical dysfunction, and we do not imply that VIP-IN hypoactivity is the main driver of HD symptoms. Rather, our experiments identify a potential leverage point for restoring circuit balance. VIP-IN activation may promote cortical plasticity and re-engage corticostriatal communication, which is disrupted early in HD^32^. Supporting this idea, cortical stimulation has been shown to improve corticostriatal plasticity and motor function in HD models^7^. This indicates that enhancing cortical adaptability can reactivate impaired corticostriatal synaptic dynamics and thereby ameliorate basal ganglia circuit dysfunction, a testable prediction that future in vivo striatal recordings can directly evaluate. Moreover, as our readout reflects somatic calcium rather than spiking directly, future cellular compartment-resolved measurements will be required to parse dendritic versus somatic contributions.

VIP-IN activity is sensitive to behavioural states, and, through their disinhibitory function, these cells contribute to the modulation of cortical gain and plasticity^12,15,33–35^. Consistent with their role in regulating plasticity, we found that the behavioural benefits of VIP-IN stimulation persisted for at least several days beyond the stimulation period. These observations suggest that the activation of VIP-INs opens the ‘gate’ for synaptic plasticity mechanisms required for motor learning through their disinhibitory role. The lasting benefits of VIP-IN stimulation suggest that the potential therapeutic applications of this approach may not require constant stimulation over long periods of time.

An endogenous mechanism of mediating VIP-IN activity regulation includes cholinergic input from basal forebrain projections, which are known to drive VIP-IN-mediated disinhibition during motor learning^12^. Cholinergic signalling is essential to facilitate synaptic plasticity and motor adaptation, processes that rely on VIP-IN-mediated modulation of excitatory output. In HD, early cholinergic deficits^36,37^ may disrupt this modulatory pathway, potentially contributing to VIP-IN dysfunction. Consistent with a broader modulation, we observed VIP-IN hypoactivity across cortical areas during motor behaviour. Nevertheless, our VIP-IN activation in the motor cortex was sufficient to improve motor behaviour. It is possible that targeting non-motor symptoms may require VIP-IN stimulation in other areas.

Our results suggest that VIP-INs may be a critical target for intervention in HD. Their uniform reduction in activity in HD seen in our experiments makes them feasible targets for artificial activity manipulation. Our data indicate that transient stimulation can produce effects that outlast the period of stimulation, consistent with a plasticity-based mechanism rather than a requirement for chronic modulation. In an intriguing contrast, our previous study has shown that inactivation of SST-INs increases excitatory plasticity but impairs learning in wild-type mice^38^. Thus, a simple reduction in inhibition is not always beneficial. VIP-IN stimulation may instead enhance plasticity while maintaining the specificity required for proper learning. In addition, the relatively slow accumulation of intranuclear mHTT inclusion bodies in VIP-INs and their less pronounced transcriptomic alterations than principal neurons^39,40^ may further argue for their suitability as a therapeutic target. More broadly, our results underscore the therapeutic potential of targeting specific neuron subtypes to rebalance neural circuit dynamics. It is important to note, however, that this circuit-level strategy is unlikely to provide a permanent repair and is also not expected to halt neurodegeneration; we view it as complementary to approaches targeting mHTT and related molecular pathways. Our work illustrates how cell-type-specific intervention can realign cortical dynamics and improve function, offering a principled path towards behavioural improvements in HD and potentially other disorders marked by identifiable circuit motifs.

## Methods

### Animals

All animal procedures were performed in accordance with guidelines set forth and protocols approved by the UCSD Institutional Animal Care and Use Committee and the US National Institutes of Health, as well as by the Government of Upper Bavaria, Germany (animal protocols 55.2-1-54-2532-168-2014, 55.2-1-54-2532-19-2015, 55.2-2532.Vet_02-20-05 and 55.2-2532.Vet_02-19-83). R6/2 mice^16^ transgenic for the 5′ end of the human huntingtin gene were obtained from Jackson Laboratories (stock no. 002810) and maintained by crossing R6/2 males to F1 C57BL/6–CBA females. Knock-in zQ175DN^19,20^ mice were obtained from Jackson Laboratories (stock no. 029928) and maintained on a C57BL/6 background. The presence of the transgene or knock-in was verified by PCR with the following primers: R6/2: forward, 5′CCGCTCAGGTTCTGCTTTTA-3′, reverse, 5′-TGGAAGGACTTGAGGGACTC-3′. zQ175DN: forward, 5′- GCGGGCTTATACCCCTACAG-3′, reverse, 5′-TCCAGGACAGCCAGAGCTAC-3′. CAG repeat length was determined by Laragen for all experimental groups. Spontaneous behavioural experiments on the wheel were performed at the Max Planck Institute for Biological Intelligence, and motorized ladder experiments were performed at the UCSD. Separate batches of R6/2 mice were used for these two sets of experiments, and the CAG repeat lengths were different between these two groups (202 ± 13 and 157 ± 6 for the Max Planck Institute and UCSD, respectively, mean ± s.d.), which led to different speeds of disease progression. Therefore, the stages of disease progression were matched across these batches of mice by monitoring their body weights (Extended Data Figs. 1a and 5b). Specifically, for R6/2 mice at the Max Planck Institute, early, middle and late stages were defined as postnatal days 49–56, 57–69 and 70–84, respectively. For R6/2 mice at the UCSD, early, middle and late stages corresponded to postnatal days 40–47, 48–55 and 56–65, respectively. Movement metrics were not used to define stages, avoiding circular logic. CAG repeat length for zQ175DN mice was 165 ± 5. Mice were group housed in cages with standard bedding in a temperature-controlled room (approximately 21 °C) with a reversed 12-h light–12-h dark cycle. Mice were allowed ad libitum access to food and water. Both male and female mice were used for all experiments.

### Surgery

Surgical procedures were performed as previously described^26,41,42^. In brief, 3.5-week-old R6/2 mice or 5-month-old zQ175DN mice were anaesthetized with an intraperitoneal injection of ketamine–xylazine (130 and 8 mg kg^−1^ body weight, respectively) and a low dose of isoflurane (0.5% with constant flow rate of 1 l min^−1^ at 0.1 bar). After reaching a deep plane of anaesthesia, enrofloxacin (10 mg kg^−1^) and dexamethasone (5 mg kg^−1^) were injected subcutaneously to prevent infection and brain swelling, respectively.

For imaging experiments, a craniotomy (4 mm in diameter) was performed, as previously described^26,41,42^, over the right caudal forelimb area of M1 centred at 0.5 mm anterior and 1.5 mm lateral from bregma. For imaging S1 (0 mm anterior and 2 mm lateral) and V1 (3 mm posterior and 3 mm lateral) cortices, a 5-mm craniotomy spanning S1 and V1 was performed. For imaging and manipulating cortical inhibitory neurons, viruses (calcium sensors: AAV9-hSyn-FLEX-jGCaMP7f or AAV2/1-hSyn-FLEX-GCaMP6f, titre of approximately 10^12^ vg ml^−1^; opsin or control: AAV5-Syn-FLEX-rc[ChR-tdT] or AAV2/1-CAG-FLEX-tdT, titre of approximately 10^13^ vg ml^−1^; Addgene) were injected into the caudal forelimb area of M1 (or S1 or V1) using a beveled glass pipette (inner diameter of approximately 12–25 µm). Each injection consisted of an approximately 200 nl volume centred at a depth of approximately 400 µm below the pial surface. Three injections, separated by at least 500 µm horizontally, were performed in each craniotomy. For imaging CStr neurons, retrograde virus (rgAAV-hSyn-jGCaMP8s, titre of approximately 10^13^ infecting units per ml; Addgene) was injected into the dorsolateral striatum at 0.5 mm anterior and 3.17 mm lateral from bregma. Two injections, each consisting of an approximately 300 nl volume were performed at a 22° angle and at 2.2 mm and 2.0 mm below the pial surface, respectively. After virus injections, the glass pipette was left in place for 5 min to avoid backflow. Following injections, a round coverslip (VWR) was implanted into the craniotomy and affixed to the skull using histoacryl glue (B.Braun) and dental acrylic cement.

For longitudinal optogenetic stimulation experiments, viruses (AAV5-Syn-FLEX-rc[ChR-tdT] or AAV2/1-CAG-FLEX-tdT, titre of approximately 10^13^ vg ml^−1^; Addgene) were injected at two sites per hemisphere through small burr holes. Each injection consisted of an approximately 200 nl volume. Injection sites were at 0.5 anterior, ±1.5 lateral and 2.0 anterior, ±1.3 lateral from bregma. Fibre optic cannulas (Doric; core diameter of 600 µm and 0.22 NA diffuser tip) were implanted at a 15° angle onto the cortical surface of both hemispheres at 1.0 anterior,  ± 1.0 lateral from bregma.

A custom-built head bar was glued and cemented to the skull to allow stable head fixation. An analgesic (buprenorphine (0.1 mg kg^−1^) or carprofen (5 mg kg^−1^)) was injected approximately 1 h before the end of the surgery to manage postoperative pain. Following surgery, mice were administered daily with Baytril, dexamethasone and analgesic for up to 3 days to manage postoperative infection, swelling and pain, respectively.

### Behaviour

#### Handling and training

At the age of 5 weeks (R6/2) or 6 months (zQ175DN), mice were handled on 4 consecutive days for 10 min until they were familiarized with the trainer and routinely ran from hand to hand. In the subsequent behavioural task training, mice got adjusted to the experimental setup and head fixation. Training sessions (30 min each, 2 and 4 consecutive days for ladder and wheel, respectively) were performed in the dark with an IR light source for the camera. Thus, mice were never trained for more than several weeks, minimizing the possibility that the motor cortex disengages due to long-term training^43,44^.

#### Motorized ladder task

Head-fixed mice were positioned onto a custom-built, circular ladder (diameter of 19 cm, rung spacing of 1 cm), adapted from the KineMouse Wheel (https://hackaday.io/project/160744-kinemouse-wheel) and motorized by an electric DC motor (12 V, 60 rpm). Trial structure was controlled using BPod (v0.5). In each trial, 8 s of ladder rotation (speed of approximately 10 mm s^−1^) were preceded by a 1-s auditory cue (3 kHz). Trials were separated by a variable 6–8-s inter-trial interval. The auditory cue (= trial start) was indicated by an approximately 30-ms IR LED flash, facilitating the alignment of trials during video analysis.

#### Wheel paradigm

Head-fixed mice were placed on a freely rotating wheel (KineMouse Wheel, diameter of 19 cm, continuous surface)^45^. No cues or trial structure were provided and mice typically showed alternating active and inactive behavioural states. Running wheel motion was captured by a rotary encoder (1,000 CPR, 60-Hz sampling rate). Speed and directionality of the wheel were decoded online using a Teensy 3.2 processor board running custom written Arduino code, adapted from Janelia Open Science Laboratory Tools (https://www.janelia.org/open-science/encoder-interface-for-mouse-treadmill).

#### Behavioural videography

For both ladder task and wheel paradigm, two orthogonal views of the mouse were captured simultaneously through a mirror mounted at 45° inside the ladder and wheel. Mouse behaviour was tracked at 60–100 Hz with an IR-sensitive video camera (USB 2.0, 1/3″CMOS, 744 × 480 pixels; 8 mm M0814MP2 1.4–16 C, 2/3″, megapixel c-mount objective; The Imaging Source) and custom software (Input Controller, The Imaging Source).

### In vivo two-photon imaging

Imaging during the motorized ladder task was performed using a commercial two-photon microscope (MOM, Sutter Instrument) equipped with a ×16/0.8-NA objective (Nikon) and a Ti:Sapphire excitation laser (Mai Tai, Spectra-Physics) tuned to 925 nm. Images (512 × 512 pixels, approximately 1 µm per pixel) were recorded at approximately 29 Hz for the duration of the behavioural session (approximately 12 min, 40–50 trials). Frame times were recorded and synchronized with behavioural recordings (videography).

Imaging during the wheel paradigm was performed using a commercial two-photon microscope (B-Scope, ThorLabs) equipped with a ×16/0.8-NA objective (Nikon) and an InsightDS+ laser (Spectra-Physics) tuned to 925 nm. Image acquisition was controlled through ThorImage 4.0 software. Images (768 × 768 pixels, 0.702 µm per pixel) were recorded at approximately 10 Hz for the duration of the behavioural session (10 min per field of view (FOV), 4–5 FOVs per session). Frame times were recorded and synchronized with behavioural recordings (videography, rotary encoder) using the ThorSync software.

Laser power at the objective was controlled with a Pockel’s cell (Conoptics) and ranged between 10 and 50 mW for all experiments. Coordinates for imaging areas, relative to bregma, were as follows: 0.5 mm anterior and 1.5 mm lateral for M1; 0 mm anterior and 2 mm lateral for S1; and 3 mm posterior and 3 mm lateral for V1. Depth of FOVs below the cortical surface was between 120 and 330 µm for VIP-INs, 120 and 550 µm for SST-INs, 160 and 530 µm for PV-INs and 130 and 530 µm for CStr neurons. For layer-specific analysis in M1, FOVs at a depth shallower and deeper than 400 µm below the cortical surface were defined as L2/3 and L5, respectively. For longitudinal repositioning, an epifluorescent image was acquired in the first imaging session to capture vasculature, and *xyz* coordinates provided by the microscope stage were documented for each FOV. The average projection two-photon image of 100 frames was used as a reference in later sessions and the *z*-plane was carefully adjusted to maximally match the imaging plane to the reference image.

### Optogenetics

For data in Figs. 3 and 4, 12 mice were used for both behaviour and imaging (included in both Figs. 3 and 4), and 27 mice were used only for behaviour (included only in Fig. 4). Imaging on post-opto days (sessions 10 and 11) was not done for technical reasons, as some FOVs became cloudy and some animals could not be maintained for follow-up. Sessions 10 and 11 were already near the end of the lifespan of R6/2 mice (approximately 10 weeks), so it was not feasible to extend these experiments further.

For simultaneous in vivo two-photon imaging and optogenetic activation of VIP-INs during the ladder task, light from a red diode laser (Oxxius LBX-638-HPE, 638 nm) was delivered through a bifurcated silica fibre optic patch cord (Doric; core diameter of 400 µm and 0.22 NA) and through implanted light diffusing fibre optic cannulas (Doric; core diameter of 600 µm and 0.22 NA, diffuser tip). One cannula was implanted over the left M1, and another was placed close to the objective illuminating the area under the cranial window. To avoid interference between stimulation and imaging, each pulse of the optogenetic light was synchronized with the resonant scanner, delivering a sub-pulse of light at the turnaround of the scanner (an 18-µs sub-pulse every 56 µs). In a small number of titration experiments (Extended Data Fig. 8; *n* = 2 VIP–Cre control mice and *n* = 2 VIP–Cre::R6/2 mice), the effective power was varied as: 0.07, 0.2, 0.4, 0.7, 1.2 and 3.7 mW, measured at the tip of cannula. These pulses were delivered at the frequency of 25 Hz with a 50% duty cycle. On the basis of these titration experiments, the rest of the experiments used the power of approximately 2-mW sub-pulses for effective approximately 0.7-mW pulses (again at 25 Hz with a 50% duty cycle). Each session consisted of 50 trials, and light stimulation was applied in 40% of trials, starting at ladder onset and terminating at 1 s after the ladder movement offset.

For optogenetic behavioural experiments without imaging, red laser light was delivered through light diffusing fibre optic cannulas implanted over M1 of both hemispheres, without the sub-pulsing described above. We tested 0.7 mW (same as imaging experiments above) and 3.7 mW in separate cohorts of mice. Stimulation was applied at 25 Hz with a 50% duty cycle, starting from the auditory cue and ladder onset to 1 s after ladder offset for 0.7-mW and 3.7-mW cohorts, respectively.

In all optogenetic experiments, to avoid visual effects of the stimulation light, mice were presented with a red-masking LED light in all trials.

### Image analysis

#### ROI identification and signal extraction

A combination of Suite2P and Cellpose^46,47^ software was used to generate regions of interest corresponding to individual neurons and to extract their fluorescence. ROI classifications by the automatic classifier were further refined by manual inspection. The time-varying baseline of a fluorescence trace (*F*_0_) was estimated using a custom-written MATLAB code by smoothing inactive portions of the trace using a previously described iterative procedure^5^. In brief, this process identified the active and inactive portions of trace, removing active portions and using the LOESS-smoothed inactive portions (interpolated across active periods) to estimate the time-varying baseline. The normalized Δ*F/F*_0_ trace was then calculated, where Δ*F* was found by subtracting the baseline trace from the raw trace, and *F*_0_ was the calculated time-varying baseline. A calcium activity event trace was constructed, which was zero except for frames with detected events, as previously described^41^. To match neurons across multiple imaging sessions, universally unique identifiers were assigned to individual neurons (ROIMatchGUI (https://github.com/sonjablumenstock/ROIMatchGUI)), followed by manual confirmation and corrections after automated detection.

#### Classification of modulated neurons

Movement-modulated neurons were classified as previously described^41^. In brief, the dot product of the binarized ladder movement trace (movements versus non-movements) and Δ*F/F*_0_ was calculated for each ROI. This value was compared with the dot products when shuffling the movement periods 1,000 times (or 5,000 times in the case of opto trials). Actual values above the 97.5 percentile of the shuffled distribution were classified as movement active, and actual values below the 2.5 percentile were classified as movement suppressed. All other cells were considered indiscriminately active. In optogenetics + imaging experiments, we classified the responses of individual neurons to stimulation. To this end, we compared mean Δ*F/F* values across light and no-light trials using Welch’s *t*-test. Resulting *P* values were corrected for multiple comparisons using the Benjamini–Hochberg false discovery rate procedure (*P* < 0.05). Neurons were classified as significantly increased or decreased in activity on the basis of the sign of the mean difference (ΔΔ*F/F* light − no light). For the classification of neurons modulated by spontaneous active or inactive behaviours, the mean Δ*F/F* in a pre-active or pre-inactive (−2 to −0.2 s before transition) and active or inactive (0–3 s) window was compared, respectively, using a Welch’s *t*-test. Cells with significantly higher or lower activity in the active or inactive window (one-sided *P* < 0.05) were labelled active or suppressed, respectively.

#### Quantification of movement modulation

Movement-related neuronal modulation (ΔΔ*F/F*_0_) in the ladder task was quantified for each neuron as the difference between mean Δ*F/F*_0_ during movement (0–8 s relative to movement onset) and mean Δ*F/F*_0_ during the pre-movement period (−1 to 0 s).

#### Relationship between movement quality and neural activity

The quality of movements on individual trials was quantified from videos using front-paw directness (*R*^2^), stride autocorrelation and hindpaw dragging time. Metrics were averaged across forelimbs where applicable, then *z*-scored across all trials; dragging values were inverted so higher scores indicated better performance. A composite behaviour score was obtained by averaging *z*-scores. Mean population Δ*F*/*F* during the movement period of each ladder trial was extracted from all recorded neurons, and relationships between behavioural composite and population activity were analysed by genotype using *X*-binned summaries (bin width of 0.3). In addition, for an animal-by-animal analysis, composite scores and mean movement-period population Δ*F*/*F* were averaged per mouse within stage, and association was assessed using Spearman rank correlation.

### Behaviour classification

For deep-learning-assisted classification of innate behaviours from raw videos, we used DeepEthogram (DEG)^21^. A single DEG model was trained in an iterative manner. On the basis of our visual observations of mouse behaviour, we defined a set of behaviour classes of interest: locomotion, grooming, rest (an inactive state with both forelimbs resting on the wheel surface), sit (an inactive state with both forelimbs resting above the wheel surface), twitch (short, fast forelimb twitches) and chew (jaw movements, including licking). An initial model was trained on approximately 10,000 manually labelled frames from a total of six exemplary videos of control and R6/2 mice of different ages. Model predictions of the initial and a few additional videos were corrected manually and used for retraining the model for the next iteration. A total of 19 training iterations was performed on a final number of 66 videos, until all behavioural classes of interest were detected with high F1 scores > 0.7. The model also identified shake (a full-body twitching behaviour), which occurred in 1.1% of frames. However, shake almost always occurred concurrently with other behavioural classes and was excluded from further analysis.

#### Voluntary behaviour-related activity

To estimate the activity of individual neurons at the transition between active and inactive behaviours, we only considered neurons with a non-zero number of calcium events detected in a given session. The activity for each neuron (*z*-scored Δ*F*/*F*_0_) was aligned to the transition between behavioural classes. We considered locomotion and grooming, classified using DEG as active motor behaviours, whereas rest and sit classes jointly represented the inactive behavioural periods. We only considered transitions between classes that were at least 3 s long to avoid contamination of activity related to other behaviours. Chew and twitch generally did not meet this criterion and their transitions were not considered for this analysis. The activity of each neuron was averaged across all individual transitions. The total number of neurons slightly differed between the two transition types due to the exclusion of transitions that occurred near the beginning or end of a session and the fact that some transitions (for example, active periods continuing until the end of a recording) could not be captured in both directions. For analysis of population activity during identified behavioural classes, we averaged each neurons activity (Δ*F*/*F*_0_) across all episodes of a classified behaviour. We did not consider chew for this analysis, as it was rare (2.6% of frames) and almost never occurred in isolation without another concurrent behaviour (0.005% of frames).

#### Relationship between locomotion speed and neural activity

For analysing relationships between locomotion speed and IN activity, Δ*F*/*F*_0_ from individual INs were aligned to the onset of locomotion bouts detected from DEG behaviour annotations. For each bout, mean *z*-scored Δ*F*/*F*_0_ and mean locomotion speed was computed in the 3 s before (baseline) and 5 s after (response) movement onset. We then calculated ΔΔ*F*/*F*_0_ and corresponding Δspeed (response − baseline) for each locomotion bout. Data were binned by Δspeed (20 mm s^−1^ bins; speeds > 200 mm s^−1^ were grouped into one bin) and averaged per mouse and bin. Bins containing 4 or more bouts per mouse and 2 or more mice per genotype were retained.

### Gait analysis

#### Data preprocessing

We used Python 3.8 and relevant libraries to process and analyse behavioural data. To extract limb movement trajectories from behavioural videos, we used DeepLabCut (DLC, v2.3)^17^. A single DLC model was trained using video frames from across a sample of experiments. Limb movements were captured from two perspectives (side and bottom). Optical distortion through the mirror was corrected by aligning *x* coordinates of side and bottom perspectives. Corrected *x* coordinates from both perspectives, detected at more than 95% DLC likelihood, were averaged. Trial information captured with BPod (v0.5) was aligned, and experimental metadata was added to each session. To preserve the essential motion characteristics while filtering out high-frequency noise, we used a Butterworth filter with a normalized cut-off removing frequencies from paw trajectories that were greater than one-tenth of the Nyquist frequency (that is, half the video frame rate).

#### Hindlimb dragging analysis

We calculated the average Euclidean distance from left forelimb to right hindlimb and from right forelimb to left hindlimb and normalized the distance by the mouse body weight^48^. To calculate the time spent dragging, the cumulative time for which the normalized forelimb–hindlimb distance exceeded 2 s.d. above the control population mean was calculated for each trial (Extended Data Fig. 1c).

#### Autocorrelation analysis

To analyse regularity in gait patterns, we autocorrelated forelimb trajectories in each individual ladder trial. To this end, we centred the trajectories around zero for each trial, removing offsets. We used the statsmodels ACF package to compute the autocorrelation function for the *x* position of each forelimb, measuring self-similarity over lags up to 5 s in each 8 s trial. The maximum peak of the autocorrelation function was determined. A peak was considered significant if it exceeded the upper bound of the 95% confidence interval, indicating significantly rhythmic behaviour in the respective trial. The fraction of significantly autocorrelated trials was determined by dividing the number of significant trials by the total number of trials.

#### Fourier analysis

To capture movement frequencies during the gait cycle, we processed the *x* coordinate trajectories of forelimbs through Fourier transformation. In short, we converted the *z*-scored trajectory of each forelimb in each trial from the time domain to the frequency domain using the scipy fft function. The power spectrum was then computed as the squared magnitude of the Fourier coefficients, representing the energy associated with each frequency component. Average power spectra were obtained by calculating the mean power within 0.13-Hz bins. The power spectrum was normalized by dividing each binned power by the maximum power, scaling the values to a range between 0 and 1, allowing comparison across sessions and animals. The maximum of the normalized power spectrum was used to extract the dominant stride frequency per session and animal.

#### Linear regression analysis

To analyse the directness of strides, we used linear regression on isolated stride phases. The *x* coordinates of zero-centred forelimb trajectories of individual trials were segmented into swing and stride phases using the peakdetect library. We defined a minimum difference in the signal required to identify extrema in the trace (5 mm). On the basis of these extrema points, the trace was segmented and labelled swing and stance depending on whether the trace was rising or falling between extrema, respectively. We used linear regression on each swing trajectory to determine the *R*^2^ score, measuring the ‘directness’ of the trajectory.

### Histology

Mice were transcardially perfused with 0.1 M sodium phosphate buffered saline (PBS) followed by 4% paraformaldehyde (wt/wt) in 0.1 M PBS. Brains were removed and postfixed in 4% paraformaldehyde overnight at 4 °C. Following postfixing, whole brains were stored in 30% sucrose in 0.1 M PBS at 4 °C for 2–3 days. Coronal sections (50 µm thick) containing the motor cortex were then prepared using a Leica SM 2000R sliding microtome. Floating sections were washed in 0.1 M PBS 3 × 5 min, mounted on slides using CC Mount (Sigma-Aldrich) and allowed to cure overnight before imaging.

### Statistics

Statistical tests were selected on the basis of data distributions and significance was set at *P* < 0.05. For neuronal activity analysis, we averaged the activity of a neuron over trials or behavioural epochs in a given session. Longitudinal behavioural measures were averaged on the session level. All evaluated datasets were tested for normality, and non-parametric tests were used throughout the article where appropriate. No statistical methods were used to pre-determine sample sizes, but our sample sizes are similar to those reported in previous publications^26,41^.

To compare two or more groups (for example, genotypes, disease stage, and time before and after behaviour onset), mixed-effects models were used to account for the nested structure of the data and minimize effects introduced by inter-animal variability^49,50^. For statistical estimation of non-normally distributed datasets, we used an Aligned Rank Transform (ART) variant of mixed-effects models using the ARTool packaged from R, which allows non-parametric mixed-effects model testing. Multiple comparisons were corrected using the two-stage false discovery rate method.

Two-factor mixed-effects models for estimating mouse body weight across disease progression (Extended Data Figs. 1a and 5b) were constructed as follows:

$$y \sim \mathrm{group}\,\mathrm{identity}+\mathrm{week}+\mathrm{genotype}:\mathrm{week}+(1|\mathrm{animal})$$

with fixed main effect terms for group identity (control and R6/2) and mouse age in weeks, a fixed interaction term for the interaction between group identity and week, and a random effect term grouped by animal. If a significant interaction was detected, post-hoc pairwise Mann–Whitney *U*-tests were performed between groups for each week.

Two-factor mixed-effects models for estimating behaviour performance and activity levels across disease stages (Figs. 1d,e,g and 2f–h and Extended Data Figs. 1d,e and 4b,c,e) were constructed as follows:

$$y \sim \mathrm{group}\,\mathrm{identity}+\mathrm{timepoint}+\mathrm{genotype}:\mathrm{timepoint}+(1|\mathrm{animal})$$

with fixed main effect terms for group identity (control and R6/2) and timepoint (early, middle and late), a fixed interaction term for the interaction between group identity and timepoint, and a random effect term grouped by animal. If a significant interaction was detected, post-hoc pairwise Mann–Whitney *U*-tests were performed between groups for each disease stage.

Similarly, to compare population average activity aligned to ladder task trials or behaviour transitions (Figs. 1j,l,n,p, 2c–e and 3e,g,j,l,m and Extended Data Figs. 2b,d,f,j,n,r, 4g,i, 7c,d and 8h,j,k), the models were constructed as follows:

$$y \sim \mathrm{group}\,\mathrm{identity}+\mathrm{time}\,\mathrm{bin}+\mathrm{genotype}:\mathrm{time}\,\mathrm{bin}+(1|\mathrm{animal})$$

with fixed main effect terms for the group identity (genotype (control and R6/2), or trial type (no light or light) or timepoint (middle and late), depending on the comparison) and 2-s time bins (−2 to 0, 0–2, 2–4, 4–6, 6–8, 8–10 and 10–12 s relative to ladder onset or −2 to 0, 0–2 and 2–4 s relative to behaviour transition), the interaction term between group identity and time bin, and a random effect term grouped by animal. If a significant interaction was detected, post-hoc pairwise Mann–Whitney *U*-tests were performed between groups for each time bin.

Two-factor mixed-effects models for quantifying changes in movement modulation across experimental groups and timepoints (Extended Data Fig. 8l) were constructed as follows:

$$\begin{array}{l}y \sim \mathrm{group}\,\mathrm{identity}+\mathrm{timepoint}+\mathrm{group}\,\mathrm{identity}\\ \,:\mathrm{timepoint}+(1|\mathrm{animal})\end{array}$$

with fixed main effect terms for group identity (Ctrl-ChR, Ctrl-tdT, R6/2-ChR and R6/2-tdT) and timepoint (middle and late), the interaction term between group identity and timepoint, and a random effect term grouped by animal. One-factor mixed-effects models for quantifying within-group changes in movement modulation across timepoints (Extended Data Fig. 8l) were constructed as follows:

$$y \sim \mathrm{timepoint}+(1|\mathrm{animal})$$

with a fixed main effect term for timepoint (middle and late) and a random intercept grouped by animal. Analyses were restricted to no-light trials.

For the subset of longitudinally tracked neurons (Extended Data Fig. 8m), we additionally accounted for repeated measures within cells:

$$y \sim \mathrm{timepoint}+(1|\mathrm{animal})+(1|\mathrm{cell})$$

with random intercepts for animal and cell universally unique identifier.

To compare behavioural performance across task learning, genotype and actuator groups (tdT and ChR), we used separate two-factor mixed effects models for control and R6/2 mice of the form:

$$\begin{array}{l}y \sim \mathrm{actuator}+\mathrm{session}\,\mathrm{number}+\mathrm{actuator}\\ \,:\mathrm{session\_number}+(1|\mathrm{animal})\end{array}$$

with fixed main effect terms for actuator (tdT and ChR) and session number, the interaction term between actuator and session number, and a random effect term grouped by animal (Fig. 4b–e and Extended Data Fig. 10b–e). For Fig. 4f and Extended Data Fig. 11b, the fixed main effect was trial type (light and no light). If a significant interaction was detected, post-hoc pairwise Mann–Whitney *U*-tests were performed between actuator groups for each session number.

To compare neuronal activity as a function of behavioural performance or locomotion speed (Extended Data Figs. 3b,c and 6a–c), we fitted a two-factor linear mixed model of the form:

$$y \sim {\rm{a}}{\rm{c}}{\rm{t}}{\rm{u}}{\rm{a}}{\rm{t}}{\rm{o}}{\rm{r}}+{\rm{b}}{\rm{e}}{\rm{h}}{\rm{a}}{\rm{v}}{\rm{i}}{\rm{o}}{\rm{u}}{\rm{r}}\,{\rm{b}}{\rm{i}}{\rm{n}}+{\rm{a}}{\rm{c}}{\rm{t}}{\rm{u}}{\rm{a}}{\rm{t}}{\rm{o}}{\rm{r}}:{\rm{b}}{\rm{e}}{\rm{h}}{\rm{a}}{\rm{v}}{\rm{i}}{\rm{o}}{\rm{u}}{\rm{r}}\,{\rm{b}}{\rm{i}}{\rm{n}}+(1|{\rm{a}}{\rm{n}}{\rm{i}}{\rm{m}}{\rm{a}}{\rm{l}})$$

with fixed main effect terms for genotype (control and R6/2), binned behavioural measure (behaviour score: 0.3 bin size; locomotion: Δspeed bins of 25 mm s^−1^), their interaction and a random effect term grouped by animal. If a significant interaction was detected, post-hoc pairwise Mann–Whitney *U*-tests were performed between groups for each behaviour bin.

One-factor mixed-effects models for comparing behavioural performance in stimulated R6/2 mice during late stimulation sessions (sessions 8 and 9) versus post-sessions (sessions 10 and 11; Extended Data Fig. 10f) were constructed as follows:

$$y \sim \mathrm{session}\,\mathrm{period}+(1|\mathrm{animal})$$

with a fixed main effect term for session period (late and post) and a random intercept grouped by animal. Because one stimulated R6/2 mouse died after session 9, paired late-versus-post analyses were restricted to animals with data in both periods. For comparisons between stimulated and unstimulated mice in post sessions, two-sided Welch’s *t*-tests were used.

For animal-by-animal analyses relating behavioural performance to population activity (Extended Data Fig. 3d), behavioural composite scores and mean movement-period population Δ*F*/*F*_0_ were averaged per mouse within disease stage, and associations were assessed using Spearman rank correlation.

All statistical test results are summarized in Supplementary Data Table 1.

### Reporting summary

Further information on research design is available in the Nature Portfolio Reporting Summary linked to this article.

## Online content

Any methods, additional references, Nature Portfolio reporting summaries, source data, extended data, supplementary information, acknowledgements, peer review information; details of author contributions and competing interests; and statements of data and code availability are available at 10.1038/s41586-026-10671-9.

## Supplementary information

Supplementary Table 1 Complete statistical output for all experiments, including p-values from aligned rank transform and linear mixed-effects models across figures and extended data. Entries specify tested factors, interactions, and experimental conditions, providing a full reference for all reported statistical comparisons.

Reporting Summary

Peer Review File

## Data Availability

Source data are provided with this paper, as well as on Zenodo (10.5281/zenodo.20750675)^51^. Additional datasets supporting the current work are available from the corresponding authors (S.B., I.D. and T.K.) on reasonable request.

## Code availability

The code used in this study is available via Zenodo (10.5281/zenodo.20750675)^51^ or from the corresponding authors (S.B., I.D. and T.K.) on request.
