# Supplementary material for: Restoring cortical disinhibition improves Huntington’s disease phenotypes
Source: Nature. 2026 Jul 1;655(8125):1262–70. doi: 10.1038/s41586-026-10671-9 (PMC13421345; doi:10.1038/s41586-026-10671-9)
Supplement: Supplementary file 2 — Reporting Summary [file 41586_2026_10671_MOESM2_ESM.pdf]

nature portfolio

Corresponding author(s): Sonja Blumenstock, Irina Dudanova, Takaki Komiyama

Last updated by author(s): May 5, 2026

Reporting Summary

Nature Portfolio wishes to improve the reproducibility of the work that we publish. This form provides structure for consistency and transparency in reporting. For further information on Nature Portfolio policies, see our Editorial Policies and the Editorial Policy Checklist.

Statistics

For all statistical analyses, confirm that the following items are present in the figure legend, table legend, main text, or Methods section.

n/a	Confirmed	
☐	☒	The exact sample size (n) for each experimental group/condition, given as a discrete number and unit of measurement
☐	☒	A statement on whether measurements were taken from distinct samples or whether the same sample was measured repeatedly
☐	☒	The statistical test(s) used AND whether they are one- or two-sided<br>*Only common tests should be described solely by name; describe more complex techniques in the Methods section.*
☐	☒	A description of all covariates tested
☐	☒	A description of any assumptions or corrections, such as tests of normality and adjustment for multiple comparisons
☐	☒	A full description of the statistical parameters including central tendency (e.g. means) or other basic estimates (e.g. regression coefficient) AND variation (e.g. standard deviation) or associated estimates of uncertainty (e.g. confidence intervals)
☐	☒	For null hypothesis testing, the test statistic (e.g. F, t, r) with confidence intervals, effect sizes, degrees of freedom and P value noted<br>*Give P values as exact values whenever suitable.*
☒	☐	For Bayesian analysis, information on the choice of priors and Markov chain Monte Carlo settings
☒	☐	For hierarchical and complex designs, identification of the appropriate level for tests and full reporting of outcomes
☒	☐	Estimates of effect sizes (e.g. Cohen's d, Pearson's r), indicating how they were calculated

Our web collection on statistics for biologists contains articles on many of the points above.

Software and code

Policy information about availability of computer code

Data collection	Two-photon imaging was performed using ThorImage (v3.4 and 4.0) and ScanImage (v4) software running on MATLAB (2011b, Mathworks). Continuous videography was aquired at 60-100 Hz via a IC Capture (v2.5). Behavioral and imaging data were synchronized using Ephus (legacy version from original publication) software running on MATLAB (2012a) or ThorSync software (Thorlabs).
Data analysis	Analysis of two-photon data was performed using a combination of previously published code (MATLAB) from the lab and additional custom code (Python, v3.6 - 3.9) to fit the needs of the current experiments.<br>Calcium image processing was performed with suite2p (v0.10), pose estimation with DeepLabCut (v2.0) and voluntary behavior was classified using DeepEthogram (v0.1). Data preprocessing workflow was managed using Snakemake (v5). Python packages used for analysis include numpy, pandas, xarray, scipy, statsmodels, scikit-learn , plots were generated using matploblib and seaborn. Matching of individual neurons across multiple imaging sessions was performed with ROIMatchGUI (https://github.com/sonjablumenstock/ROIMatchGUI)

For manuscripts utilizing custom algorithms or software that are central to the research but not yet described in published literature, software must be made available to editors and reviewers. We strongly encourage code deposition in a community repository (e.g. GitHub). See the Nature Portfolio guidelines for submitting code & software for further information.

Data

Policy information about availability of data

All manuscripts must include a data availability statement. This statement should provide the following information, where applicable:

- Accession codes, unique identifiers, or web links for publicly available datasets
- A description of any restrictions on data availability
- For clinical datasets or third party data, please ensure that the statement adheres to our policy

Data and code are deposited in a CodeOcean capsule, available at: https://codeocean.com/capsule/7629434/tree

Research involving human participants, their data, or biological material

Policy information about studies with human participants or human data. See also policy information about sex, gender (identity/presentation), and sexual orientation and race, ethnicity and racism.

Reporting on sex and gender	No human participants or human data were used in this study.
Reporting on race, ethnicity, or other socially relevant groupings	*Please specify the socially constructed or socially relevant categorization variable(s) used in your manuscript and explain why they were used. Please note that such variables should not be used as proxies for other socially constructed/relevant variables (for example, race or ethnicity should not be used as a proxy for socioeconomic status).* *Provide clear definitions of the relevant terms used, how they were provided (by the participants/respondents, the researchers, or third parties), and the method(s) used to classify people into the different categories (e.g. self-report, census or administrative data, social media data, etc.)* *Please provide details about how you controlled for confounding variables in your analyses.*
Population characteristics	*Describe the covariate-relevant population characteristics of the human research participants (e.g. age, genotypic information, past and current diagnosis and treatment categories). If you filled out the behavioural & social sciences study design questions and have nothing to add here, write "See above."*
Recruitment	*Describe how participants were recruited. Outline any potential self-selection bias or other biases that may be present and how these are likely to impact results.*
Ethics oversight	*Identify the organization(s) that approved the study protocol.*

Note that full information on the approval of the study protocol must also be provided in the manuscript.

Field-specific reporting

Please select the one below that is the best fit for your research. If you are not sure, read the appropriate sections before making your selection.

☒ Life sciences ☐ Behavioural & social sciences ☐ Ecological, evolutionary & environmental sciences

For a reference copy of the document with all sections, see nature.com/documents/nr-reporting-summary-flat.pdf

Life sciences study design

All studies must disclose on these points even when the disclosure is negative.

Sample size	No formal statistical tests were run to pre-determine sample size. However, sample sizes were based on pilot experiments and similar to those reported in previous own publications. The intrinsic failure rate of the experiments, the success rate of imaging cells across learning and disease progression, and signal-to-noise ratio of the imaging sensor were critical factors in initial estimates.
Data exclusions	Imaging data were only excluded from the data used in this study if optical access to the imaged cells showed commonly cited signs of deterioration, i.e. if visibility was considerably lower during or across imaging sessions so as to prevent analysis. For longitudinal imaging and behavior datasets, only mice that completed all experimental sessions were included in the analysis.
Replication	Imaging during voluntary and skilled movement motor behaviors were conducted in two different labs and in separate batches of mice. The same functional changes in cortical interneurons during motor behavior were observed independently and were thus considered successful replications. For all experiments, trends in data were evaluated based on pilot experiments and reproduction of the results was considered successful if the same trends were observed in samples taken from different experimental cohorts of mice. All of the data presented in this study adhered to this process.
Randomization	Animals used in this study were not selected based on any other prerequisite features other than general animal wellbeing appropriate for the respective age and genotype (e.g. normal grooming and social behavior, no obvious infections etc.) When possible, equal numbers of mice from each cage were used for each experimental group (genotype or actuator) so as to minimize batch effects of each cohort.
Blinding	Experimenters were not blinded to the genotype of mice, due to the fact that the obvious phenotype of R6/2 mice (e.g. weight loss, reduced motor activity) makes blinding during experiments impossible. However, both imaging and behavioral data (ROI detection, behavior tracking and classification) were entirely processed by unbiased, automated algorithms (suite2p, DeepLabCut, DeepEthogram) and decision

parameters accessible during 2-photon imaging experiments were: 1) optimized for data quality only, 2) conceptually many steps removed from the central parameters used in this study (e.g. activity during various quiet and active behaviors) making it extremely unlikely that the data could be biased in one predictable direction, and 3) impossible to use as a priori prediction criteria due to disease progression or learning-related changes. Thus, knowledge of conditions is extremely unlikely to produce bias in our measurements.

Reporting for specific materials, systems and methods

We require information from authors about some types of materials, experimental systems and methods used in many studies. Here, indicate whether each material, system or method listed is relevant to your study. If you are not sure if a list item applies to your research, read the appropriate section before selecting a response.

Materials & experimental systems

n/a	Involved in the study
☒	☐ Antibodies
☒	☐ Eukaryotic cell lines
☒	☐ Palaeontology and archaeology
☐	☒ Animals and other organisms
☒	☐ Clinical data
☒	☐ Dual use research of concern
☒	☐ Plants

Methods

n/a	Involved in the study
☒	☐ ChIP-seq
☒	☐ Flow cytometry
☒	☐ MRI-based neuroimaging

Animals and other research organisms

Policy information about studies involving animals; ARRIVE guidelines recommended for reporting animal research, and Sex and Gender in Research

Laboratory animals	All animal procedures were performed in accordance with guidelines set forth and protocols approved by the UCSD Institutional Animal Care and Use Committee and the National Institutes of Health. Mice (mus musculus, males and females, 6-12 weeks old) were used. R6/2 mice transgenic for the 5' end of the human huntingtin gene were obtained from Jackson Laboratories and maintained by crossing R6/2 males to F1 C57Bl6/CBA females (either obtained by Jackson Laboratories (UCSD) or bred in-house (MPIB). Mice were group housed in cages with standard bedding in a temperature controlled room (~21ºC) with a reversed 12hr light/12hr dark cycle. Mice were allowed ad libitum access to food and water. All experiments were performed during the dark cycle.
Wild animals	This study did not involve wild animals.
Reporting on sex	Both male and female mice were used and wherever possible, we selected balanced numbers of male and female control and R6/2 mice for each experiment. Overall, male and female mice account for about half of the total mice used in this study and all findings are based on data from both sexes.
Field-collected samples	This study did not involve animals collected from the field.
Ethics oversight	UCSD Institutional Animal Care, National Institutes of Health and Use Committee and Government of Upper Bavaria, Germany (animal protocols 55.2-1-54-2532-168-2014, 55.2-1-54-2532-19-2015, 55.2-2532.Vet_02-20-05, and 55.2-2532.Vet_02-19-83)

Note that full information on the approval of the study protocol must also be provided in the manuscript.

Plants

Seed stocks	*Report on the source of all seed stocks or other plant material used. If applicable, state the seed stock centre and catalogue number. If plant specimens were collected from the field, describe the collection location, date and sampling procedures.*
Novel plant genotypes	*Describe the methods by which all novel plant genotypes were produced. This includes those generated by transgenic approaches, gene editing, chemical/radiation-based mutagenesis and hybridization. For transgenic lines, describe the transformation method, the number of independent lines analyzed and the generation upon which experiments were performed. For gene-edited lines, describe the editor used, the endogenous sequence targeted for editing, the targeting guide RNA sequence (if applicable) and how the editor was applied.*
Authentication	*Describe any authentication procedures for each seed stock used or novel genotype generated. Describe any experiments used to assess the effect of a mutation and, where applicable, how potential secondary effects (e.g. second site T-DNA insertions, mosiacism, off-target gene editing) were examined.*
