# Supplementary material for: Restoring cortical disinhibition improves Huntington’s disease phenotypes
Source: Nature. 2026 Jul 1;655(8125):1262–70. doi: 10.1038/s41586-026-10671-9 (PMC13421345; doi:10.1038/s41586-026-10671-9)
Supplement: Supplementary file 3 — Peer Review File [file 41586_2026_10671_MOESM3_ESM.pdf]

nature portfolio

Peer Review File

Restoring cortical disinhibition improves Huntington's disease phenotypes

Corresponding Author: Professor Takaki Komiyama

Any redactions in this file are there to maintain patient confidentiality, the confidentiality of unpublished data, or to remove third-party material.

This file contains all reviewer reports in order by version, followed by all author rebuttals in order by version.

Version 0:

Reviewer comments:

Referee #1

(Remarks to the Author)
This study by Blumenstock et al. seeks to determine the cortical microcircuit deficits in Huntington's disease mouse model. There are several strengths. The characterization of the movement is strong, relying on both a ladder task that requires learning and forced movement as well as a spontaneous movement assay where they apply machine learning algorithm to isolate behavioral events. The analyses are convincing. The imaging approach is powerful because it allows longitudinal characterization of cell type specific activity.

The main finding is exciting, revealing cell-type specific alterations associated with movements in the mouse model. The idea that stimulating the interneurons can partially reverse the deficits and improve performance is also exciting.

The circuit dissection is simplistic. More can be done to understand the subtypes of corticostriatal neuron, and also whether how special is this VIP-SST pathway in the pathophysiology relative to other elements in the cortical microcircuit. Mechanistically it also does not tell us how the genetic alterations may cause the disturbance in the VIP and SST interneurons.

Major comment:

Are the deficits seen in Fig. 1i-k due simply to reduced neuronal activity associated with reduced or perturbed movement, and that it is simply movement-related activity dynamics rather than reflecting anything for the pathophysiology? From the data shown in Fig. 2c-d, there seems to be HD-specific differences but then these don't track progression where significant differences were observed across all stages.

Minor comment:

How are early, middle, and late disease stage defined? It is not very clear from the method. Is it from the movement? Or from body weight? If it is defined by movement, but then it is used to analyze movements to show that movement across different stages are different, the logic is circular.

A premise is that the cortical circuits are important. However there are also well cited studies showing that a wide range of movements do not involve the motor cortex in rodents (e.g., PMID 25892304). What aspects of movements (e.g., hindlimb dragging, stride regularity, cadence etc.) in this motorized ladder task require the motor cortex?

There are two main types of corticostriatal neurons – pyramidal tract and intratelencephalic. Are the deficits observed specific to one subtype or to both?

The animals likely exhibit different types of walking patterns, so aggregating to show the average like Fig. 1i-k may obscure

these potentially interesting trial-specific features.

Line 100: The cortical layer for the imaged neurons should be specified.

Where is the imaging location in terms of the somatotopy?

There is a disconnect here where stimulating the VIP interneuron leads very specifically more excitable dendrites via SST interneurons. However, the imaging of CSPN is done at the soma to show that the firing activity was corrected. Is the dendrite important for this restoration? What if PV interneurons were suppressed, which can also leads to higher firing, or what if the CSPNs are directly excited, in both cases to match the control?

Is it learning or execution? What if the opto is applied during learning but not used during the test – to show it is strictly learning, or alternatively what if opto is not applied during learning but used during the test – to see if it is execution?

Are the same neurons in the same field of view imaged across sessions? This seems to be the case shown in Ext. Data Fig. 3. There is a missed opportunity to look at longitudinal changes at the single cell level, which is possible with this elegant imaging approach. The data analysis seems to mostly aggregate the neurons, instead of considering session-to-session changes in the individual cells?

Referee #2

(Remarks to the Author)
The study by Blumenstock et al. used a mouse model of Huntington's disease (HD), R6/2 mice, to investigate the changes in motor related responses in several types of cortical neurons, including corticostriatal projection neurons (CSPNs), VIP and SST interneurons, accompanying movement deficits. Following characterization of activity differences between R6/2 mice and control mice, authors investigated the therapeutic potential of optogenetics in addressing motor deficits in R6/2 mice. They found that optogenetic activation of VIP interneurons restored activity levels of CSPNs. In parallel, activation of VIP interneurons also improved motor functions in R6/2 mice with motor deficits.

This study represents an important step forward in understanding the role of cortical circuits in HD-related motor dysfunction. The authors demonstrated the feasibility of cell-type specific interventions to improve motor function in HD mouse models.

However, several challenges remain, and several strengths and limitations should be considered.

Major points

1. Characterization of the cortical neuron responses is preliminary. While this study focuses on the CSPNs and specific subtypes of interneurons (VIP and SST), it is important to determine the relationship between the activity of these cortical neurons and the motor behavior being examined. Fig. 1i-k only show the overall activity differences between R6/2 and control mice without taking into account the relationship between neuronal activity and motor behaviors. While the study uses comprehensive measurements of movement kinematics to illustrate the motor deficits of R6/2 mice, the relationships between these kinematics and the cortical neuronal subtypes have not been analyzed. What is the single-neuron level heterogeneity in coding for the movement kinematics? How single-neuron responses or information coding were altered across the HD progression? Most of analyses are based on grand averaging of population responses, without taking advantage of the single-neuron resolution imaging data. While this study emphasizes the technical advantage of tracking single-neuron level activity across the disease progression, the longitudinal changes in neuronal responses to different motor kinematics over the progression have not been analyzed.

2. Evidence for causality is incomplete. A key result in this study is that optogenetic activation of VIP interneurons improved motor learning and mitigated motor deficits of R6/2 mice. In parallel, the authors showed that activation of VIP interneurons disinhibited SST interneurons, which in turn elevated the activity level of CSPNs. These results imply that VIP interneurons and their downstream SST and CSPNs are causally involved in the motor behaviors under examination and their dysfunction may lead to the progression of HD symptoms. It is therefore important to directly test the causal links. Would prolonged inactivation of VIP interneurons lead to any progression of motor deficits?

3. Specificity in brain regions and neural circuits needs further verification. The authors broadly referred to the neocortex and striatum as key regions involved in HD, but these are vast areas that mediate a wide range of functions beyond motor control. While the authors only examined the activity and effect of manipulation in M1, it is important to determine whether the observed activity changes in R6/2 mice are specific to M1. Could other cortical areas also exhibit decreased activity levels in VIP neurons? This is particularly relevant when considering the authors' hypothesis that deficits in cholinergic modulatory inputs might be the cause of altered VIP activity, as the BF ACh system projects extensively to numerous cortical and subcortical areas. Relatedly, would the effect on motor behavior following VIP neuron manipulation specific for M1? It seems control experiments in other cortical regions may be necessary.

This study presents the disinhibitory circuit (VIP-SST-CSPN) as a major neural substrate and therapeutic target for HD related motor deficits. But this presentation together with the recurring simplistic circuit diagram is suggestive of an oversimplified picture. VIP interneurons not only target SST but also other types of inhibitory neurons, and SST interneurons not only target CSPNs but also other pyramidal neurons and interneurons. In addition, while the corticostriatal circuits are implicated in HD, the abnormal striatal activity would in turn influence a wide range of cortical activity via thalamic inputs.

The authors should at least discuss about these nuances and how the circuit motif identified in this study could fit into the broader networks of HD related dysfunctions.

4. This study demonstrates the effectiveness of optogenetic stimulation of VIP-INs in normalizing CSPN activity and improving motor performance in HD mouse model. The authors stated that the results “could inform future therapies aimed at modulating the activity of distinct neuronal populations to alleviate symptoms”. However, it is important to demonstrate the robustness of such intervention. How sensitive are the effects to the parameters of the stimulation protocol, e.g., laser power, duration, stimulation location, and virus expression range etc.? The current study only provides a single stimulation condition. It would be more informative if the authors could at least test different levels of laser power.

Minor points

1. Some of the statistical details are not clear. For example, in Fig. 2c,d, it is not clear whether the plots are from a single neuron, or from averaging of all neurons? If from population averaging, how many neurons? Fig. 2e,f shows grand averaging without providing number of neurons or behavioral sessions.

2. For the effects on cortical neuron activity following VIP activation (Fig. 3), the authors again only showed grand averaging. What is the neuronal level heterogeneity? How many CSPNs showed increased/decreased/no-change activity?

3. The circuit diagram shown in Fig. 3 can be misleading. The activity change in SST neurons following VIP activation was not examined. It is unclear whether the effects of VIP activation on CSPNs were through disinhibition of SST neurons.

4. It appears that the effects of VIP stimulation were not restricted within the stimulation period (Fig. 4f), suggesting that the optogenetic stimulation may have a lasting effect on the circuits. Could such lasting effect be found in the neuronal data (Fig. 3)?

Referee #3

(Remarks to the Author)
The manuscript by Blumenstock and colleagues describes an interesting set of experiments in which the activity of three types of M1 cortex neurons in a mouse model of Huntington’s disease (HD) were longitudinally monitored and correlated with movement. Using in vivo Ca2+ imaging, the authors found that during cued and spontaneous movement the activity of VIP GABAergic interneurons and corticostriatal pyramidal neurons was depressed in R6/2 mice, whereas that of SST interneurons was elevated. The authors then show that optogenetic stimulation of VIP interneurons restores movement-related activity in the M1 circuit and improves motor function in R6/2 mice. These experiments are elegant, nicely described and illustrated. That said, I have a number of concerns surrounding the relevance of the model for HD, the interpretation of the data and the mechanisms underlying the optogenetic ‘restoration’ of circuit function.

Major concerns:

• At best, the R6/2 mouse is a model of juvenile HD, not adult-onset HD. The pathology accompanying over-expression of mutant exon 1 is not the same as in other HD models, like the more widely embraced knock-in models. The authors need to discuss the limitations this model imposes on the conclusions about HD pathogenesis and symptomatic treatment.

• It would have strengthened the paper had in vivo imaging data been gathered in the early stages of the pathophysiology of the R6/2 mice to determine if VIP interneuron activity (and that of the rest of the cortical circuit) was normal initially. Had the knock-in model, which progresses more slowly, been used, these pre-manifest measurements would have been easier to make.

• Based upon the plots in Fig. 1, the abnormal activity of SST and CSPB neurons was seen in a distinct subset of neurons (~30%). The use of parametric statistics to summarize population behavior obscures this apparent heterogeneity. The authors should explore alternative means of presenting their data and discuss this heterogeneity. The pyramidal neurons projecting to the ipsilateral striatum belong to both the IT and PT classes. Is it possible that they are differentially affected? A simple experiment to narrow the pyramidal neurons sampled would be to look at neurons infected by a contralateral injection of virus; this would restrict the sample to IT neurons.

• Is the alteration in pyramidal neuron activity seen in the HD model specific to those projecting to the striatum?

• How was the optogenetic stimulation protocol determined? How does the frequency/duration/intensity of VIP interneuron stimulation affect the circuitry and behavior? Furthermore, as the activity readout was a GCaMP Ca2+ signal, how do the authors know that optogenetic stimulation of VIP neurons normalized spiking? I suppose this inference is based upon the ability to ‘correct’ CSPN activity levels (again measured with GCaMP).

• The most fascinating part of the study is the observation that VIP stimulation improved motor learning and ameliorated motor symptoms in R6/2 mice. But there are some puzzling aspects of these experiments. The authors report that the CSPN activity was restored on trials in which the VIP interneurons were stimulated but not on trials when they were not stimulated. But the improvement in motor performance was independent of whether the light was on or off. Doesn’t this argue that the improvement in behavior does not depend upon normalization of CSPN activity?

• If the VIP optogenetic stimulation affected motor learning on the ladder, did the improvement in movement persist for days, weeks or months after termination of the stimulation?

Minor concerns:

• The authors also should at least briefly discuss the relationship between the somatic Ca2+ signal and spiking, particularly given that the SST synapses are dendritic.

• How does optogenetic ‘normalization’ of CSPN activity affect functional connectivity with the striatum? At the outset, the authors talk about corticostriatal ‘disconnection’ as a key early event in HD pathophysiology.

• Was bilateral M1 stimulation necessary? Is the effect specific to M1? Does stimulation of other cortical regions projecting to the striatum have similar effects?

Version 1:

Reviewer comments:

Referee #1

(Remarks to the Author)
The authors performed a large number of new analyses to address the comments. The addition of a second knock-in HD model to address another reviewer’s comment is notable and greatly strengthens the study. I have one additional comment below.

Thank you for performing a new analysis quantifying the composite kinetic score to show that cortical dysfunction was consistent across movement qualities, suggesting that the cortical dysfunction was associated with the phenotype rather than just a product of the abnormal movements. This is not a foolproof analysis, but serves to support the author’s point and address the major comment.

One remaining question: Much of the results shown average across all individuals within a phenotype to show control versus HD model differences in interneuron activity. If we look on an individual-by-individual basis, does the maladaptive interneuron activity of an individual predict its the motor function? This is a relevant question if one wants to use the interneuron activity as a biomarker of disease progression.

Referee #2

(Remarks to the Author)

A central claim of the study is that activation of VIP-INs normalizes CSPN activity in R6/2 mice with a persistent effect. However, the support for this conclusion remains unclear for the following reasons.

At the middle time point (Fig. 3i,j), activation of VIP-INs did not bring CSPN activity in R6/2 mice closer to that in control mice, which makes the interpretation as a normalization effect difficult. In Fig. 3j, the y-axis appears to be missing a tick mark and may be incorrectly scaled, and the tick labels seems to be incorrectly positioned, making it difficult to assess the amplitude of the reported effect.

At the late time point (Fig. 3k,l), the authors stated (Line 245) that CSPN activity in stimulated R6/2 mice is statistically indistinguishable from control animals. However, the data in Fig. 3k,l are challenging to interpret because the control group activity appears lower than that shown for control mice at the middle time point, while it is difficult to tell if CSPN activity in R6/2 mice is substantially higher than at the middle time point (with peaks of mean traces both around 0.08). This would raise the possibility that the apparent effect only reflected differences in baseline and control activity rather than a true change in movement related activity in R6/2 mice.

The difference in the control data shown in Fig. 3i,j and Fig. 3k,l is confusing. From the heatmap (Fig. 3i and Fig. 3k), it visually appears that the activity in control mice at the late time point is overall lower than that at the middle time point. Are they from the same population of neurons tracked across the two time points? The presented results would be more transparent if the authors could provide representative single-neuron examples for various conditions, and make pairwise comparisons for chronically tracked neurons.

Regarding the persistent effect following VIP-IN stimulation (Fig. 4), it remains unclear to me whether it is an across-trial effect or it could last over multiple sessions or days after the stimulation has stopped. The lack of difference between interleaved light and no-light trials (Fig. 4f) only suggests that the stimulation effect could be maintained across trials. But in the two extra sessions after stimulation shown in Fig. 4c, the stride regularity was clearly decreasing without stimulation, arguing against the proposed lasting therapeutic effect after treatment. The authors may want to test the post-stimulation effects in a longer period of time to assess their persistence.

Referee #3

(Remarks to the Author)
The authors have done an excellent job of addressing my concerns. At this point, the manuscript makes a compelling, if somewhat surprising, case for the conclusions about the impact of optogenetic restoration of cortical activity on the motor deficits in HD.

Version 3:

Reviewer comments:

Referee #1

(Remarks to the Author)
Thank you for performing the additional analysis. It is interesting to see the interneuron deficits correlate with behavioral impairments. I believe this further bolster the conclusions of the paper.

I have no further comment.

I commend the authors for an excellent and detailed study, with important implications for therapeutics.

Referee #2

(Remarks to the Author)
In the second revision, the authors addressed previous comments by correcting figure errors, and providing explanations. While these improvements are noted, they do not fully resolve my previous concerns.

I previous noted that CStr activity in control animals appears to decrease from middle to late stages, raising concerns of data variability. This is a concern because the increased activity observed in R6/2 mice (Rebuttal Figure 2 d,e), which the authors attributed to the VIP-IN stimulation, could be at least partially due to a variation of activity across learning stages. The authors should rule out this possibility.

In Fig. 4, my previous concern was that there is a clear trend of decreasing stride regularity following the cessation of stimulation, which argues against the authors' claim of persistent post-stimulation effects. The authors should test this trend statistically by comparing the final stimulation session directly to the final post-stimulus session. However, the authors reported a non-significant difference by pooling the 2 sessions before and after stimulation cessation, thereby diluting this trend. If a significant decrease in stride regularity exists post-stimulation, the authors should acknowledge this result and temper their claims accordingly.

Referee #3

(Remarks to the Author)
The authors have responded constructively to my concerns. I have no further issues.

Version 4:

Reviewer comments:

Referee #2

(Remarks to the Author)
In this revision, the authors have provided quantifications addressing my previous comments. I have no further comments.

Overall this study should be praised for linking cell-type specific local cortical circuits to therapeutic implications for a neurological condition.

Open Access This Peer Review File is licensed under a Creative Commons Attribution 4.0 International License, which permits use, sharing, adaptation, distribution and reproduction in any medium or format, as long as you give appropriate credit to the original author(s) and the source, provide a link to the Creative Commons license, and indicate if changes were made.
In cases where reviewers are anonymous, credit should be given to 'Anonymous Referee' and the source.
The images or other third party material in this Peer Review File are included in the article’s Creative Commons license, unless indicated otherwise in a credit line to the material. If material is not included in the article’s Creative Commons license and your intended use is not permitted by statutory regulation or exceeds the permitted use, you will need to obtain permission directly from the copyright holder.
To view a copy of this license, visit https://creativecommons.org/licenses/by/4.0/

We thank the reviewers for their thoughtful evaluation and constructive suggestions. Several strengths were highlighted across the reports, including our longitudinal, cell-type–specific two-photon imaging, rigorous kinematic characterization of behavior, and the demonstration that targeted VIP-interneuron activation can improve motor performance in HD mice. We appreciate the reviewers' recognition that the study addresses a timely question about cortical circuit mechanisms and therapeutic leverage points in HD.

In revising the manuscript, we have incorporated new analyses and experiments that directly address the major concerns. Some of the major additions are:

1. Using another mouse model of HD (the knock-in zQ175DN), we have confirmed that hypoactivity of VIP-INs and corticostriatal neurons is consistent across models.
2. We have shown that behavioral improvements of VIP-IN stimulation
    a. last for days after stimulation, and
    b. robust across stimulation powers.
3. We have shown that hypoactivity of VIP-INs is consistent across cortical areas.
4. We have added data from PV-INs that show hyperactivity in HD, similarly to SST-INs.

Below we provide detailed, point-by-point responses.

Referee #1 (Remarks to the Author):

This study by Blumenstock et al. seeks to determine the cortical microcircuit deficits in Huntington's disease mouse model. There are several strengths. The characterization of the movement is strong, relying on both a ladder task that requires learning and forced movement as well as a spontaneous movement assay where they apply machine learning algorithm to isolate behavioral events. The analyses are convincing. The imaging approach is powerful because it allows longitudinal characterization of cell type specific activity.

The main finding is exciting, revealing cell-type specific alterations associated with movements in the mouse model. The idea that stimulating the interneurons can partially reverse the deficits and improve performance is also exciting.

The circuit dissection is simplistic. More can be done to understand the subtypes of corticostriatal neuron, and also whether how special is this VIP-SST pathway in the pathophysiology relative to other elements in the cortical microcircuit. Mechanistically it also does not tell us how the genetic alterations may cause the disturbance in the VIP and SST interneurons.

Major comment:

Are the deficits seen in Fig. 1i-k due simply to reduced neuronal activity associated with reduced or perturbed movement, and that it is simply movement-related activity dynamics rather than reflecting anything for the pathophysiology? From the data shown in Fig. 2c-d,

there seems to be HD-specific differences but then these don't track progression where significant differences were observed across all stages.

We agree that movement differences are a key potential confound. To directly address this issue, in the revision we explicitly quantified trial-to-trial variability in movement quality and related it to cortical activity. We quantified the movement quality on individual trials using a composite kinematic score and found that cell-type-specific cortical dysfunction was consistent across movement qualities (Extended Data Fig. 3a–c and Extended Data Fig. 6). Thus, cortical dysfunction is not a result of movement deficits and instead reflects HD-specific circuit phenotypes.

Minor comment:

How are early, middle, and late disease stage defined? It is not very clear from the method. Is it from the movement? Or from body weight? If it is defined by movement, but then it is used to analyze movements to show that movement across different stages are different, the logic is circular.

Stages were defined by postnatal age bins and corroborated by body weight and CAG repeat length (Extended Data Fig. 1a). Movement metrics were not used to define stages, avoiding circular logic. We now state this explicitly in Results and Methods (Results p. 3, lines 92–94, Methods p. 2, lines 43–51).

A premise is that the cortical circuits are important. However there are also well cited studies showing that a wide range of movements do not involve the motor cortex in rodents (e.g., PMID 25892304). What aspects of movements (e.g., hindlimb dragging, stride regularity, cadence etc.) in this motorized ladder task require the motor cortex?

We clarify that this task requires precise limb placement, grasping, and rapid adaptation, functions often attributed to M1 circuits. Our training/testing timeline samples early learning (R6/2 mice never reach "expert" performance), minimizing the chance that cortex disengages after long-term training. Importantly, the manipulation of M1 VIP-INs affects the behavior studied, providing a causal link between task performance and M1 activity. We added relevant citations in the Methods (p. 3, lines 100–102) and one a clarifying sentence in Results (p. 2, lines 90–92).

There are two main types of corticostriatal neurons – pyramidal tract and intratelencephalic. Are the deficits observed specific to one subtype or to both?

To address this issue, we separately analyzed CStr neuron activity in L2/3 and L5, where IT and PT neurons are enriched, respectively. HD-related hypoactivity was comparable in both layers (Extended Data Fig. 2a-d), suggesting that both projection classes are affected. We note this in Results (p. 3, lines 125–127).

The animals likely exhibit different types of walking patterns, so aggregating to show the average like Fig. 1i-k may obscure these potentially interesting trial-specific features.

As described in our reply to the major comment above (Extended Data Fig. 3a-c), we have analyzed trial-level variability in movement quality. Despite broad overlap in behavior-quality distributions between genotypes, VIP-IN and CStr activity remained significantly lower in R6/2 mice across all quality bins.

Line 100: The cortical layer for the imaged neurons should be specified.
Where is the imaging location in terms of the somatotopy?

We apologize for the omission. We now specify the imaging depths for each class (VIP, SST, PV, CStr) and the location of (caudal forelimb area) in the Methods. We added a brief statement in the Results (p. 3, lines 113–115) and figure legend (Fig. 1).

There is a disconnect here where stimulating the VIP interneuron leads very specifically more excitable dendrites via SST interneurons. However, the imaging of CSPN is done at the soma to show that the firing activity was corrected. Is the dendrite important for this restoration? What if PV interneurons were suppressed, which can also leads to higher firing, or what if the CSPNs are directly excited, in both cases to match the control?

We agree that our data do not directly distinguish dendritic from somatic contributions to the observed rescue. Our interpretation does not depend on dendrite-specific mechanisms. Although VIP interneurons are classically positioned to relieve dendritic inhibition through suppression of SST interneurons, VIP activation can also influence PV interneurons and thereby increase the overall excitability of cortical principal neurons. Consistent with this broader circuit engagement, we observe enhanced movement-related activity of PV interneurons in R6/2 mice (new results presented in Figures 1m-n, 2e, h and Extended Data Fig 5g). We therefore interpret the effect of VIP stimulation as a general cortical disinhibition, not limited to SST-mediated dendritic compartments. We have clarified this interpretation in the revised Discussion (p. 7, lines 318–326).

Is it learning or execution? What if the opto is applied during learning but not used during the test – to show it is strictly learning, or alternatively what if opto is not applied during learning but used during the test – to see if it is execution?

We thank the reviewer for the opportunity to further clarify this critical point. We believe our manipulation enhances learning. Our optogenetic experiments directly address this distinction: within sessions, light and no-light trials were behaviorally indistinguishable (Fig. 4f), whereas performance improved across sessions in R6/2-ChR mice. Additionally, we now provide new results that show that the improvements persisted for days post-stimulation (Fig. 4b–e, “Post”), consistent with learning-related changes rather than transient facilitation. We clarify this interpretation in Results (Figs. 3–4; Extended Data Figs. 8–9).

Are the same neurons in the same field of view imaged across sessions? This seems to be the case shown in Ext. Data Fig. 3. There is a missed opportunity to look at longitudinal changes at the single cell level, which is possible with this elegant imaging approach. The data analysis seems to mostly aggregate the neurons, instead of considering session-to-session changes in the individual cells?

Indeed, the same neurons were imaged longitudinally across early, middle, and late disease stages. We extended our analysis to explicitly track single neurons across sessions. These longitudinal analyses are now presented in Extended Data Fig. 2e-t, where matched VIP neurons (in M1, S1, and V1; panels a–l) and CStr neurons (panels m–p) were classified as movement-active (MA), movement-suppressed (MS), or indiscriminately active (IA). In addition to average population activity, we visualize transitions of the same neurons between functional classes across timepoints. Tracked neurons revealed that R6/2 VIP-IN and CStr neurons were more likely to lose MA identity and become MS/IA across timepoints than ctrl (Extended Data Fig. 2h, l, p,t).

We extended this analysis to the activity of IN types during spontaneous behaviors (Extended Data Fig. 5e–g), tracking individual VIP-, SST-, and PV INs across timepoints. VIP-INs showed a similar loss of MA identity across timepoints as observed in the ladder task experiments, while the fraction of MA neurons was increased in both SST- and PV-INs (Extended Data Fig. 5e-g).

These new experiments and analyses directly address single-cell heterogeneity and longitudinal dynamics.

Referee #2 (Remarks to the Author):

The study by Blumenstock et al. used a mouse model of Huntington’s disease (HD), R6/2 mice, to investigate the changes in motor related responses in several types of cortical neurons, including corticostriatal projection neurons (CSPNs), VIP and SST interneurons, accompanying movement deficits. Following characterization of activity differences between R6/2 mice and control mice, authors investigated the therapeutic potential of optogenetics in addressing motor deficits in R6/2 mice. They found that optogenetic activation of VIP interneurons restored activity levels of CSPNs. In parallel, activation of VIP interneurons also improved motor functions in R6/2 mice with motor deficits.

This study represents an important step forward in understanding the role of cortical circuits in HD-related motor dysfunction. The authors demonstrated the feasibility of cell-type specific interventions to improve motor function in HD mouse models.

However, several challenges remain, and several strengths and limitations should be considered.

Major points

1. Characterization of the cortical neuron responses is preliminary. While this study focuses on the CSPNs and specific subtypes of interneurons (VIP and SST), it is important to determine the relationship between the activity of these cortical neurons and the motor behavior being examined. Fig. 1i-k only show the overall activity differences between R6/2 and control mice without taking into account the relationship between neuronal activity and motor behaviors. While the study uses comprehensive measurements of movement kinematics to illustrate the motor deficits of R6/2 mice, the relationships between these kinematics and the cortical neuronal subtypes have not been analyzed. What is the single-neuron level heterogeneity in coding for the movement kinematics? How single-neuron responses or information coding were altered across the HD progression? Most of analyses are based on grand averaging of population responses, without taking advantage of the single-neuron resolution imaging data. While this study emphasizes the technical advantage of tracking single-neuron level activity across the disease progression, the longitudinal changes in neuronal responses to different motor kinematics over the progression have not been analyzed.

We now analyze neuronal activity with behavior quality controlled in two complementary ways. First, by quantifying movement qualities on individual trials in the ladder task, we found that hypoactivity of VIP-IN and CStr neurons is consistent across movement qualities (Extended Data Fig. 3a–c). Second, comparing VIP- and SST-IN activity after matching running speed across genotypes shows that genotype effects persist (Extended Data Fig. 6). Taken together, VIP-IN and CStr neuron hypoactivity with SST-IN hyperactivity remain after accounting for kinematics and locomotion speed; they reflect HD-specific circuit imbalance, not mere movement differences.

To test for longitudinal changes in neuronal responses, we extended our analysis to explicitly track single neurons across sessions. These longitudinal analyses are now presented in Extended Data Fig. 2, where matched VIP neurons (in M1, S1, and V1; panels e–p) and CStr neurons (panels q–t) were classified as movement-active (MA), movement-suppressed (MS), or indiscriminately active (IA). In addition to average population activity, we visualize transitions of the same neurons between functional classes across timepoints. Tracked neurons revealed that R6/2 VIP-INs and CStr neurons were more likely to lose MA identity and become MS/IA between early→late than control (Extended Data Fig. 4h, l, p, t), directly addressing single-cell heterogeneity and longitudinal dynamics.

2. Evidence for causality is incomplete. A key result in this study is that optogenetic activation of VIP interneurons improved motor learning and mitigated motor deficits of R6/2 mice. In parallel, the authors showed that activation of VIP interneurons disinhibited SST interneurons, which in turn elevated the activity level of CSPNs. These results imply that VIP interneurons and their downstream SST and CSPNs are causally involved in the motor behaviors under examination and their dysfunction may lead to the progression of HD symptoms. It is therefore important to directly test the causal links. Would prolonged inactivation of VIP interneurons lead to any progression of motor deficits?

We thank the reviewer for the opportunity to clarify this important point. We agree that our study does not show that VIP dysfunction is the main driver of HD symptoms. In fact, HD involves widespread neuronal dysfunction and degeneration and it is likely that many of these events across brain areas contribute to HD symptoms. Our study instead is designed to test a therapeutic leverage—i.e., whether augmenting VIP-IN activity can restore cortical dynamics and improve motor performance in HD—rather than to claim that VIP-IN dysfunction is the sole or primary cause of HD motor pathology. We now clarify this more explicitly in the Discussion section (p. 7, lines 326–330).

While we did not perform a VIP-IN inactivation experiment, our prior work (Ren et al., Neuron 2022) showed that suppressing VIP-INs in young healthy mice produces mild but significant motor impairments. We cite this study and note that their findings are consistent with the idea that VIP-IN activity contributes to motor control and learning, but that VIP-IN dysfunction is one component within a broader, distributed pathology in HD.

Nevertheless, our existing results provide a strong support for a functional rescue: VIP-IN activation normalizes VIP-IN and CStr neuron activity during movement (Fig. 3), and—critically—provides a lasting improvement of motor behavior that persists for days (Fig. 4), arguing for learning-related plasticity rather than a transient performance boost. These effects align with the role of VIP-INs in state-dependent disinhibition and plasticity gating.

3. Specificity in brain regions and neural circuits needs further verification. The authors broadly referred to the neocortex and striatum as key regions involved in HD, but these are vast areas that mediate a wide range of functions beyond motor control. While the authors only examined the activity and effect of manipulation in M1, it is important to determine whether the observed activity changes in R6/2 mice are specific to M1. Could other cortical areas also exhibit decreased activity levels in VIP neurons? This is particularly relevant when considering the authors' hypothesis that deficits in cholinergic modulatory inputs might be the cause of altered VIP activity, as the BF ACh system projects extensively to numerous cortical and subcortical areas. Relatedly, would the effect on motor behavior following VIP neuron manipulation specific for M1? It seems control experiments in other cortical regions may be necessary.

We agree that establishing regional scope is important. In the revision, we imaged VIP-IN activity in primary motor (M1), somatosensory (S1) and visual (V1) cortices at matched timepoints. In both S1 and V1 we observed reduced movement-related VIP activity in R6/2 mice, similarly to M1: (i) lower VIP-IN activation during movement epochs and (ii) a shift toward movement-suppressed classifications of individual neurons relative to controls (new Extended Data Fig. 2e-p; wheel paradigm transitions in Extended Data Fig. 5e-g). These cross-area findings suggest a cortex-wide modulation of VIP-INs during learning and state changes (Ren et al., Neuron 2022; Szadai et al., eLife 2022).

Despite this global dysfunction, motor performance improved with motor cortex-specific manipulation. It is possible that improving non-motor symptoms might require stimulation in other areas. We discuss this in Discussion (p. 8, lines 350–353).

This study presents the disinhibitory circuit (VIP-SST-CSPN) as a major neural substrate and therapeutic target for HD related motor deficits. But this presentation together with the recurring simplistic circuit diagram is suggestive of an oversimplified picture. VIP interneurons not only target SST but also other types of inhibitory neurons, and SST interneurons not only target CSPNs but also other pyramidal neurons and interneurons. In addition, while the corticostriatal circuits are implicated in HD, the abnormal striatal activity would in turn influence a wide range of cortical activity via thalamic inputs. The authors should at least discuss about these nuances and how the circuit motif identified in this study could fit into the broader networks of HD related dysfunctions.

The reviewer raises an important point about the broader complexity of cortical microcircuits and their interactions with subcortical networks. Our schematic was intended to highlight a circuit motif consistent with the data rather than to imply exclusivity. In the revised Discussion, we clarify that VIP-INs modulate both SST- and PV-IN populations and thus can relieve both dendritic and perisomatic inhibition onto principal neurons (p. 7, lines 318–326). Notably, our revision includes new data showing increased movement-related activity of PV-INs in R6/2 mice across our behavioral paradigms (Fig. 1m,n, Fig. 2e,h, Extended Data Fig. 5g). These findings support a model of network-level disinhibition, in which reduced VIP-IN activity disrupts multiple inhibitory pathways and results in hypoactive CStr populations.

4. This study demonstrates the effectiveness of optogenetic stimulation of VIP-INs in normalizing CSPN activity and improving motor performance in HD mouse model. The authors stated that the results “could inform future therapies aimed at modulating the activity of distinct neuronal populations to alleviate symptoms”. However, it is important to demonstrate the robustness of such intervention. How sensitive are the effects to the parameters of the stimulation protocol, e.g., laser power, duration, stimulation location, and virus expression range etc.? The current study only provides a single stimulation condition. It would be more informative if the authors could at least test different levels of laser power.

We agree that it is important to test the robustness of rescue with stimulation parameters, and now provide power titrations and two stimulation powers for behavioral rescue.
We varied the light power and measured the activity of VIP-INs during the ladder task. VIP activity scaled with power: 0.2–0.4 mW drove small activity increases, 0.7 mW restored R6/2 VIP activity to the level of unstimulated control mice, and 1.2–3.7 mW drove larger-than-physiological responses (Extended Data Fig. 8b). Based on this, we used 0.7 mW in the imaging experiments (Fig. 3). For behavior analysis, we used 0.7 mW and 3.7 mW to test the robustness across stimulation strengths (Fig. 4, Extended Data Fig. 9). Both powers improved behavior across kinematic measurements. Thus, behavioral rescue is robust across stimulation parameters.

Minor points

1. Some of the statistical details are not clear. For example, in Fig. 2c,d, it is not clear whether the plots are from a single neuron, or from averaging of all neurons? If from population averaging, how many neurons? Fig. 2e,f shows grand averaging without providing number of neurons or behavioral sessions.

We apologize for the confusion. We now indicate the number of neurons and sessions in all figure legends.

2. For the effects on cortical neuron activity following VIP activation (Fig. 3), the authors again only showed grand averaging. What is the neuronal level heterogeneity? How many CSPNs showed increased/decreased/no-change activity?

We thank the reviewer for this insightful comment. To address it, we now include an analysis of neuronal-level heterogeneity in Extended Data Fig. 8d,e. We calculated the changes in movement-related activity of individual neurons (light − no light) and classified them as significantly increased, decreased, or unchanged. This analysis revealed that a larger fraction of CStr neurons showed significant activity increases in R6/2 mice, demonstrating that VIP-IN activation effectively re-engages hypoactive CStr neurons rather than producing uniform changes across the population.

3. The circuit diagram shown in Fig. 3 can be misleading. The activity change in SST neurons following VIP activation was not examined. It is unclear whether the effects of VIP activation on CSPNs were through disinhibition of SST neurons.

We agree that the original schematic could imply a single-pathway mechanism. Our VIP-activation experiments in Fig. 3 were designed to test sufficiency at two readouts—VIP-INs and CStr neurons – not to isolate a single interneuron intermediary. We therefore do not claim that SST is the only route by which VIP-IN activation normalizes CStr neuron activity. Although we did not record SST-INs simultaneously during VIP-IN activation in this revision, prior work directly supports the VIP→SST link: Ren et al. (2022) showed that inhibiting VIP-INs increases SST-IN activity during motor learning, consistent with VIP-IN-mediated inhibition of SST-INs.

Given our revised Fig. 1 results—showing increased SST- and PV-IN activity in R6/2 during movement—both classes are plausible conduits for reduced net inhibition onto CStr neurons when VIP-INs are driven. To reflect this nuance, we have updated the circuit diagram in Fig. 3 and other figures to include a PV branch (perisomatic inhibition of pyramidal/CStr neurons) alongside SST (dendritic inhibition).

4. It appears that the effects of VIP stimulation were not restricted within the stimulation period (Fig. 4f), suggesting that the optogenetic stimulation may have a lasting effect on the circuits. Could such lasting effect be found in the neuronal data (Fig. 3)?

We thank the reviewer for this excellent question. This is an important observation and aligns with our interpretation that VIP-IN stimulation induces lasting, learning-related

plasticity in the circuit rather than a transient “light-on” effect. Our revised study provides additional evidence for this:

1. Behavior (Fig. 4f):
   Our original manuscript had shown that, within sessions, performance on light and no-light trials was indistinguishable across groups, indicating that stimulation did not acutely alter motor execution. However, across sessions, R6/2-ChR animals showed progressive improvements. We now provide new results showing that these gains persisted for days after stimulation ceased (sessions 10–11), consistent with a durable behavioral rescue.
2. Neural activity (Fig. 3):
   We have now performed two-photon recordings in both middle and late disease stages during longitudinal VIP stimulation.
   - VIP-INs (Fig. 3d–g): As expected for directly stimulated neurons, VIP-IN activity increased only during light trials, confirming acute activation.

   - CStr neurons (Fig. 3i–m): At the late disease stage, R6/2 mice with VIP-IN stimulation exhibited elevated CStr neuron activity compared to R6/2 controls in both light and no-light trials. This normalization of CStr neuron output in the absence of ongoing stimulation aligns with the behavioral persistence, indicating a lasting network repair rather than momentary optogenetic drive.

   Together, these data suggest that repeated VIP-IN activation promotes enduring cortical circuit rebalancing and behavioral improvement—consistent with learning-related plasticity rather than transient execution effects. We now emphasize this point in the revised Results (p. 6/7, lines 278-290) and Discussion (p. 7, lines 337–344).

Referee #3 (Remarks to the Author):

The manuscript by Blumenstock and colleagues describes an interesting set of experiments in which the activity of three types of M1 cortex neurons in a mouse model of Huntington’s disease (HD) were longitudinally monitored and correlated with movement. Using in vivo Ca2+ imaging, the authors found that during cued and spontaneous movement the activity of VIP GABAergic interneurons and corticostriatal pyramidal neurons was depressed in R6/2 mice, whereas that of SST interneurons was elevated. The authors then show that optogenetic stimulation of VIP interneurons restores movement-related activity in the M1 circuit and improves motor function in R6/2 mice. These experiments are elegant, nicely described and illustrated. That said, I have a number of concerns surrounding the relevance of the model for HD, the interpretation of the data and the mechanisms underlying the optogenetic ‘restoration’ of circuit function.

Major concerns:

• At best, the R6/2 mouse is a model of juvenile HD, not adult-onset HD. The pathology accompanying over-expression of mutant exon 1 is not the same as in other HD models,

like the more widely embraced knock-in models. The authors need to discuss the limitations this model imposes on the conclusions about HD pathogenesis and symptomatic treatment.

We agree that R6/2 is a rapid, early-onset HD model that does not recapitulate all features of the disease. We now acknowledge this explicitly in the Discussion (p. 7, lines 310–314). To address generalizability, we have performed new *in vivo* two-photon experiments in the knock-in zQ175DN mice. Please see below for details.

• It would have strengthened the paper had in vivo imaging data been gathered in the early stages of the pathophysiology of the R6/2 mice to determine if VIP interneuron activity (and that of the rest of the cortical circuit) was normal initially. Had the knock-in model, which progresses more slowly, been used, these pre-manifest measurements would have been easier to make.

To directly address this point, we have performed new experiments in the knock-in zQ175DN mice at the early HD stage (6-month-old). At this stage, zQ175DN mice showed only mild behavioral impairment in the ladder task (Extended Data Fig. 4b-e), and their spontaneous behaviors on the wheel were comparable to controls (Extended Data Fig. 7b). With *in vivo* imaging, we found that VIP-, SST-, and CStr neurons showed altered activity defects that were qualitatively similar to but milder than what we reported in R6/2 mice in both the ladder and wheel paradigms, indicating the core cortical signature is consistent across mouse models (Extended Data Fig. 4f-i and Extended Data Fig 7c-f).

• Based upon the plots in Fig. 1, the abnormal activity of SST and CSPN neurons was seen in a distinct subset of neurons (~30%). The use of parametric statistics to summarize population behavior obscures this apparent heterogeneity. The authors should explore alternative means of presenting their data and discuss this heterogeneity. The pyramidal neurons projecting to the ipsilateral striatum belong to both the IT and PT classes. Is it possible that they are differentially affected? A simple experiment to narrow the pyramidal neurons sampled would be to look at neurons infected by a contralateral injection of virus; this would restrict the sample to IT neurons.

We thank the reviewer for highlighting the heterogeneity among cortical neuron types. To better represent this variability, we now present single-cell response in heatmaps throughout the paper, and classify individual neurons as movement-active (MA), movement-suppressed (MS), or indiscriminately active (IA) (see Methods). Longitudinal tracking further shows that individual R6/2 VIP-IN neurons are more likely to lose their MA identity across stages than controls (Extended Data Fig. 2e-p).

To address potential projection-class differences, we separately analyzed CStr neurons from layer 2/3 (enriched for intratelencephalic, IT, neurons) and layer 5 (enriched for pyramidal-tract, PT, neurons). HD-related hypoactivity was comparable in both layers (Extended Data Fig. 3), suggesting that both projection classes are affected. We have incorporated this information into the revised Results (p. 3, lines 125–127).

• Is the alteration in pyramidal neuron activity seen in the HD model specific to those projecting to the striatum?

Our main dataset focuses on corticostriatal (CStr) projection neurons because the corticostriatal pathway is a primary locus of HD motor pathology. To assess whether the observed alterations are specific to this projection class, we compared CStr neuron activity across cortical layers enriched for intratelencephalic (IT, L2/3) and pyramidal tract (PT, L5) neurons. HD-related hypoactivity was comparable in both layers (Extended Data Fig. 2a-d), suggesting that the dysfunction is not restricted to a single projection class but reflects a broader deficit in cortical output.

• How was the optogenetic stimulation protocol determined? How does the frequency/duration/intensity of VIP interneuron stimulation affect the circuitry and behavior? Furthermore, as the activity readout was a GCaMP Ca2+ signal, how do the authors know that optogenetic stimulation of VIP neurons normalized spiking? I suppose this inference is based upon the ability to 'correct' CSPN activity levels (again measured with GCaMP).

We agree that it is important to test the robustness of rescue with stimulation parameters. To directly address this issue, we have expanded the section on VIP stimulation. We varied the light power and measured the activity of VIP-INs during the ladder task. VIP activity scaled with power: 0.2–0.4 mW drove a small increase in VIP activity, 0.7 mW restored R6/2 VIP activity to the level of unstimulated ctrl mice, and 1.2–3.7 mW drove larger-than-physiological responses (Extended Data Fig. 8b). Based on this, we used 0.7 mW in the imaging experiments (Fig. 3). For behavior analysis, we used 0.7 mW and 3.7 mW to test the robustness across stimulation strengths (Fig. 4, Extended Data Fig. 9). Both powers improved behavior across kinematic measurements. Thus, behavioral rescue is robust across a certain range of stimulation parameters.

We agree with the reviewer that our manipulation may not be perfectly normalizing spiking activity of cortical neurons. However, the new results show that VIP-IN stimulation is effective across stimulation strengths, indicating that perfect normalization is not necessary for lasting behavioral benefits. We now state this explicitly (p. 6, lines 275–277).

• The most fascinating part of the study is the observation that VIP stimulation improved motor learning and ameliorated motor symptoms in R6/2 mice. But there are some puzzling aspects of these experiments. The authors report that the CSPN activity was restored on trials in which the VIP interneurons were stimulated but not on trials when they were not stimulated. But the improvement in motor performance was independent of whether the light was on or off. Doesn't this argue that the improvement in behavior does not depend upon normalization of CSPN activity?

We appreciate the reviewers' insightful point, which prompted us to perform additional experiments and analyses. The behavioral and neural data together indicate that VIP-IN stimulation induces lasting circuit reorganization rather than a transient light-on effect. Within sessions, behavioral performance was similar on light and no-light trials (Fig. 4f), confirming

that the stimulation did not acutely alter motor execution. However, behavior improved progressively across sessions. We now show that the behavioral improvement persisted for days after stimulation ceased, consistent with learning-related plasticity.

We also performed longitudinal two-photon recordings (Fig. 3), which show that CStr neuron activity in R6/2-ChR mice became normalized across both light and no-light trials at the late stage, consistent with the persistent behavioral improvement. Thus, while the initial VIP-IN activation drives acute disinhibition, repeated stimulation produces durable CStr circuit normalization that supports the long-term behavioral gains. We now highlight this relationship between circuit and behavioral plasticity in the revised Results (p. 5/6, lines 243–248, p. 6/7, lines 278-290) and Discussion (p. 7, lines 337–344).

• If the VIP optogenetic stimulation affected motor learning on the ladder, did the improvement in movement persist for days, weeks or months after termination of the stimulation?

In our revised study, we recorded behavioral performance for two additional timepoints beyond the stimulation period (2 and 4 days after the last stimulation). The behavioral improvement of R6/2-ChR persisted for those sessions after the last stimulation, spanning multiple days (Fig. 4b–f). The stabilization of performance in later sessions indicates that the effect is not transient within-session facilitation but reflects lasting learning-related improvement. Future work in other HD models will assess whether these gains endure over longer timescales (weeks to months).

Minor concerns:

• The authors also should at least briefly discuss the relationship between the somatic Ca2+ signal and spiking, particularly given that the SST synapses are dendritic.

Our revised dataset shows movement-related SST-IN and PV-IN hyperactivity (Fig. 1k–n) and CStr neuron hypoactivity (Fig. 1o–p). The combined perisomatic and dendritic increase in inhibition is consistent with reduced cortical output (p. 7, lines 318–326). We caution against over-interpreting absolute firing rates and note future compartment-resolved readouts (p. 7, lines 334–336).

• How does optogenetic ‘normalization’ of CSPN activity affect functional connectivity with the striatum? At the outset, the authors talk about corticostriatal ‘disconnection’ as a key early event in HD pathophysiology.

We now discuss the idea that restoring CStr activity could re-enable corticostriatal plasticity and improve downstream activity, consistent with early “disconnection” hypotheses; we add references and frame this as a testable prediction for future in vivo striatal recordings (p. 7, lines 328–334).

• Was bilateral M1 stimulation necessary? Is the effect specific to M1? Does stimulation of other cortical regions projecting to the striatum have similar effects?

To test regional specificity, we imaged VIP-IN activity in primary motor (M1), somatosensory (S1) and visual (V1) cortices at matched timepoints. In both S1 and V1 we observed reduced movement-related VIP-IN activity in R6/2 mice, similarly to M1: (i) lower VIP-IN activation during movement epochs and (ii) a shift toward movement-suppressed classifications of individual neurons relative to controls (new Extended Data Fig. 2e-p; wheel paradigm transitions in Extended Data Fig. 5e-g). Of note, VIP-IN activity patterns in M1 were less stable across sessions in R6/2 mice than in controls, particularly between the early and middle stages, suggesting progressive loss of VIP-IN modulation in M1 in addition to global hypoactivity.

For stimulation experiments, we targeted M1, and no other cortical regions, because our study focuses on motor behaviors and the ladder task depends on motor learning. Accordingly, we used bilateral stimulation to engage the motor cortices controlling both sides of the body during locomotion. We note that for non-motor functions, stimulation of other cortical areas may be more effective. We discuss this in Discussion (p. 8, lines 350–353).

We thank the reviewers for their thoughtful evaluation and appreciate their constructive suggestions. Reviewers 1 and 3 have given positive evaluations of the revised manuscript, endorsing its publication. Reviewer 1 made one request. Reviewer 2 raised two sets of concerns. In revising the manuscript, we have incorporated new analyses that directly address these requests and concerns. Please see below our detailed, point-by-point responses.

Reviewer 1:

One remaining question: Much of the results shown average across all individuals within a phenotype to show control versus HD model differences in interneuron activity. If we look on an individual-by-individual basis, does the maladaptive interneuron activity of an individual predict its the motor function? This is a relevant question if one wants to use the interneuron activity as a biomarker of disease progression.

This is an excellent suggestion. We have performed this analysis and found that R6/2 mice with lower (i.e. more abnormal) movement-related VIP-IN activity tended to have poorer behavioral performance. This effect reached statistical significance in the late stage (Rebuttal Fig. 1). While we cannot make strong claims due to limited numbers of animals in our datasets (6 R6/2 and 4 control VIP mice), we describe this tendency in Results (p. 3, lines 129-131) and in an Extended Data Figure (Extended Data Fig. 3d).

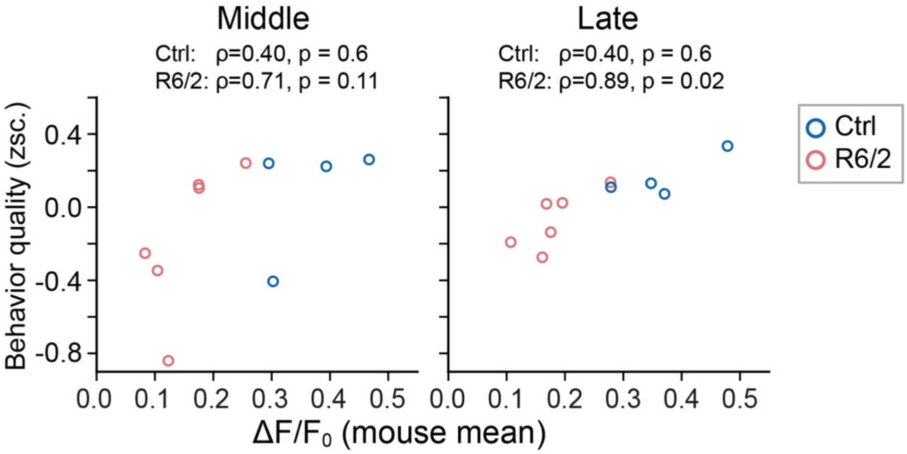

Rebuttal Figure 1 (Extended Data Fig. 3d): R6/2 mice with lower movement-related VIP-IN activity tended to have poorer behavioral performance.
Mean movement-related VIP-IN activity of each mouse is plotted against mean behavioral composite score at middle and late disease stages. Spearman correlations are indicated.

Reviewer 2:

A central claim of the study is that activation of VIP-INs normalizes CSPN activity in R6/2 mice with a persistent effect. However, the support for this conclusion remains unclear for the following reasons.

At the middle time point (Fig. 3i,j), activation of VIP-INs did not bring CSPN activity in R6/2 mice closer to that in control mice, which makes the interpretation as a normalization effect difficult. In Fig. 3j, the y-axis appears to be missing a tick mark and may be incorrectly scaled, and the tick labels seems to be incorrectly positioned, making it difficult to assess the amplitude of the reported effect.

At the late time point (Fig. 3k,l), the authors stated (Line 245) that CSPN activity in stimulated R6/2 mice is statistically indistinguishable from control animals. However, the data in Fig. 3k,l are challenging to interpret because the control group activity appears lower than that shown for control mice at the middle time point, while it is difficult to tell if CSPN activity in R6/2 mice is substantially higher than at the middle time point (with peaks of mean traces both around 0.08). This would raise the possibility that the apparent effect only reflected differences in baseline and control activity rather than a true change in movement related activity in R6/2 mice.

The difference in the control data shown in Fig. 3i,j and Fig. 3k,l is confusing. From the heatmap (Fig. 3i and Fig. 3k), it visually appears that the activity in control mice at the late time point is overall lower than that at the middle time point. Are they from the same population of neurons tracked across the two time points? The presented results would be more transparent if the authors could provide representative single-neuron examples for various conditions, and make pairwise comparisons for chronically tracked neurons.

We appreciate the reviewers' insightful comment. Part of this concern likely originated from an error in Fig. 3j, in which 2 of the y axis tick labels ('0.04' and '0.08') had been inadvertently misplaced. We sincerely apologize for the confusions caused by this error. We confirm that there were no other errors—there was no missing tick mark, and scaling was correct and consistent across Fig. 3j, 3l, and 3m. We show the corrected Figure below (Rebuttal Fig. 2a and 2b, corresponding to manuscript Fig. 3j and 3l).

Our central claim in Fig. 3 is that VIP-IN activation drives a modest increase in CStr activity in the middle stage, and this increase accumulates over sessions to be closer to normal in the late stage. This cumulative benefit is observed in the late stage even without acute light stimulation, likely due to enhanced plasticity during prior light activation.

We now include additional analyses directly demonstrating this claim.

First, we include a direct test for the acute effect of VIP-IN stimulation in the middle stage in R6/2 mice. This shows a modest but highly significant increase in CStr activity in light trials compared to no-light trials (Rebuttal Fig. 2a, corresponding to manuscript Fig. 3j).

Second, we include a direct test for the improvement of CStr activity from middle to late stages in no-light trials of VIP-IN-stimulated R6/2 mice. This test shows a significant increase in CStr activity across time in no-light trials (Rebuttal Fig. 2c and 2d, corresponding to Extended Data Fig. 8k and 8l). We have also confirmed this result in the subset of the dataset in which the same cells were longitudinally tracked (Rebuttal Fig. 2e, corresponding to Extended Data Fig. 8m), directly addressing the Reviewer request. We now clarify these points in the revised Results (p. 6, lines 229-244).

The Reviewer also pointed out that CStr activity in control animals appears to decrease from middle to late stages, raising concerns of data variability. We believe this appearance was exaggerated due to our error in the y-axis labeling in Fig. 3j, for which we again apologize. We also cannot assume that activity levels are stable over learning in control animals, because learning-related activity refinement has been well-documented. Therefore, a potential difference across learning stages in control animals is not necessarily a concern. Nevertheless, our new analysis above circumvents this issue by directly comparing the levels in R6/2 mice across treatment stages. We have also added examples of single neuron activity in Extended Data Figure 9 as requested.

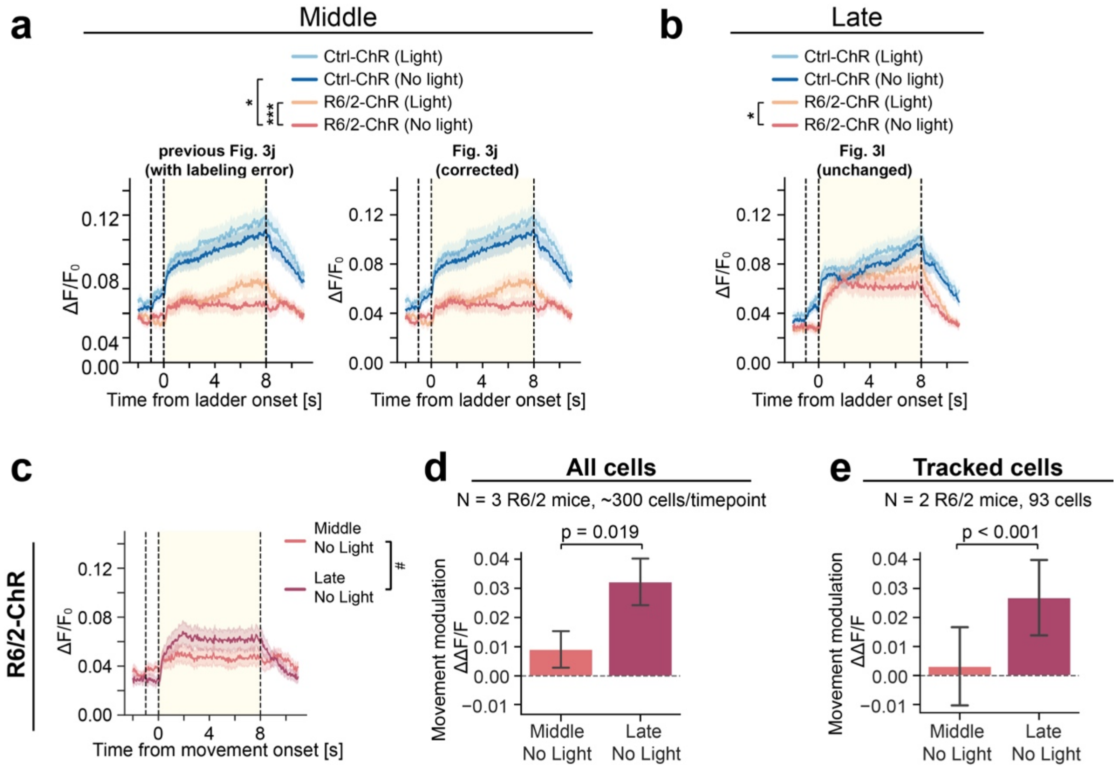

Rebuttal Figure 2 (from Fig. 3 and Extended Data Fig. 8). VIP-IN stimulation enhances movement-related activity in corticostriatal (CStr) neurons in R6/2 mice.

(**a**) Trial-averaged calcium responses ($\Delta F/F_0$) of CStr neurons aligned to ladder onset in control and R6/2 mice during the middle disease stage, shown separately for light and no-light trials; dashed lines denote cue onset, ladder onset, and ladder offset. This figure corresponds to the manuscript Fig. 3j. Light stimulation in R6/2 mice drove acute disinhibition of CStr neurons (R6/2 light vs. no-light, trial type × time bin interaction, $p < 0.001$), but the activity remained lower than in control animals (light (R6/2) vs. no-light (Ctrl): genotype main effect, $p < 0.05$). The previous submission contained error in the y-axis tick labels (left panel), which has been corrected (right panel). Aligned rank transform (ART) two-factor statistics; trial type × time bin interaction: *$p < 0.05$, ***$p < 0.001$. Mean ± s.e.m.

(**b**) Same as (a) but for the late disease stage, corresponding to the manuscript Fig. 3l. There was no error in the previous submission.

(**c**) Movement-aligned CStr activity in R6/2–ChR mice during middle vs late sessions in no-light trials, re-plotted from (a) and (b), indicating an increase in CStr activity from the middle to late stage. Aligned rank transform (ART) two-factor statistics; trial type main effect: #$p < 0.05$.

(**d**) Population-level movement modulation, defined as $\Delta F/F_0$ during movement (0–8 s) minus baseline (−1–0 s), quantified across all recorded CStr neurons in R6/2–ChR mice. Mean ± s.e.m. Linear mixed models with mice as random intercepts.

(**e**) Within-neuron changes in movement modulation between middle and late stages for longitudinally tracked CStr neurons in 2 R6/2–ChR mice. Mean ± s.e.m. Linear mixed models with mice and cells as random intercepts.

Regarding the persistent effect following VIP-IN stimulation (Fig. 4), it remains unclear to me whether it is an across-trial effect or it could last over multiple sessions or days after the stimulation has stopped. The lack of difference between interleaved light and no-light trials (Fig. 4f) only suggests that the stimulation effect could be maintained across trials. But in the two extra sessions after stimulation shown in Fig. 4c, the stride regularity was clearly decreasing without stimulation, arguing against the proposed lasting therapeutic effect after treatment. The authors may want to test the post-stimulation effects in a longer period of time to assess their persistence.

We thank the reviewer for the opportunity to clarify this point. Our central claim in Fig. 4 is that VIP-IN stimulation in R6/2 mice drives behavioral improvements that last for days after treatment cessation.

We now provide two new sets of analysis to further support this claim (Rebuttal Fig. 3, Extended Data Fig. 10f). First, we compared the performance in the 2 post-stimulation sessions (sessions 10 and 11) between VIP-IN-stimulated R6/2 mice and non-stimulated R6/2 mice. This analysis showed that the performance remained significantly better in VIP-IN-stimulated mice across all behavioral measures. Second, within the VIP-IN-stimulated R6/2 mice, we compared the last 2 stimulation sessions (sessions 8 and 9) and the 2 post-stimulation sessions (sessions 10 and 11). Across all behavioral measures, their performance remained similar after stimulation cessation, with a further improvement, though modest, in one measure (hindlimb dragging time). We clarify this in the revised Results section (p. 7, lines 280-288).

The Reviewer pointed out that there is a decreasing trend for the behavioral improvements in one of the 4 measures (stride regularity). We have found that this decreasing trend is partially attributed to one animal that died after session 9 and so did not contribute to the data in sessions 10-11 (this animal shown as open circles in the bar plots in Rebuttal Fig. 3). Nevertheless, we believe our results collectively establish that the behavioral improvements last for days after the treatment. Furthermore, we do not claim that the improvement is permanent, and we have clarified this point in the Results (p. 7, lines 288-290) and Discussion (p. 9, lines 365-367). Even though we agree with the Reviewer that a further analysis for longer periods of time could be informative, it would not have been feasible to extend this experiment beyond the 4 days after the treatment, because sessions 10-11 were already near the end of the lifespan of R6/2 mice (~10 weeks). We have added this information in the Methods section (p. 4, lines 150-152).

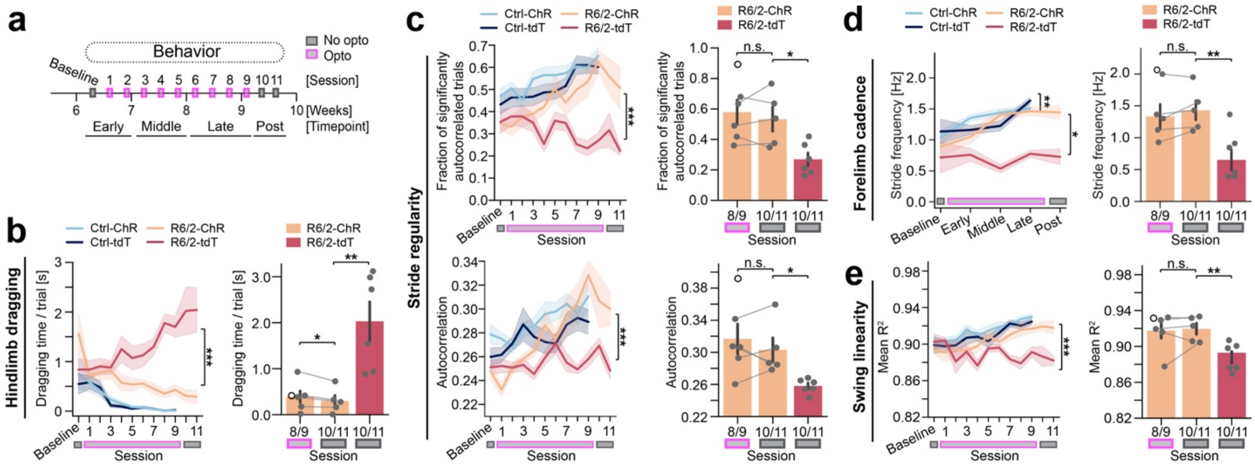

Rebuttal Figure 3 (From Fig. 4 and Extended Data Fig. 10f). **VIP-IN stimulation improves motor performance in R6/2 mice.**

(**a**) Experimental timeline. After one baseline recording, mice were trained in the ladder task with optogenetic stimulation in 40% of trials every other day for 9 sessions, followed by 2 additional behavior sessions without stimulation ('Post').

(**b**) Duration of hindlimb dragging per trial decreased with training in ctrl-ChR, ctrl-tdTomato, and R6/2-ChR mice. In R6/2-ChR mice, performance in the sessions after stimulation (sessions 10–11) remained significantly better than that of R6/2-tdT mice.

(**c**) Autocorrelation analysis of forelimb trajectories reveals increasing regularity of oscillatory gait patterns with learning: fraction of significantly autocorrelated trials (top) and maximum autocorrelation value per trial (bottom). Improvements were observed in ctrl-ChR, ctrl-tdTomato, and R6/2-ChR mice. The loss of one R6/2–ChR mouse with highly regular gait after session 9 (open circle) contributes to the apparent reduction in the 'post' sessions; paired analyses of surviving R6/2-ChR mice show no significant difference between sessions 8–9 and sessions 10–11, and their performance on sessions 10-11 is significantly better than that of R6/2-tdT mice.

(**d**) Mean peak frequencies of trial-averaged power spectra of forelimb trajectories increased over training, reflecting increased stepping cadence in control and R6/2-ChR mice.

(**e**) Regression analysis of individual forelimb strides. Average R^2 values, reflecting swing directness, increased with motor learning in control mice and R6/2-ChR mice.

Line plots (from Fig. 4) show aligned rank transform (ART) statistics; actuator × session interaction: *$p < 0.05$, **$p < 0.01$, ***$p < 0.001$. Bar graphs (from Extended Data Fig. 10f) show paired comparisons (sessions 8/9 versus 10/11) using linear mixed models with mice as random intercepts and unpaired comparisons (R6/2-ChR versus R6/2-TdT) using unpaired Welch's t-test; *$p < 0.05$, **$p < 0.01$. Mean ± s.e.m.

We thank the reviewers for evaluating our manuscript again. Reviewers 1 and 3 have given positive evaluations of the revised manuscript, endorsing its publication. Reviewer 2 has asked for further clarification on two points. Please see below our detailed, point-by-point response.

Reviewer 2:

In the second revision, the authors addressed previous comments by correcting figure errors, and providing explanations. While these improvements are noted, they do not fully resolve my previous concerns.

I previous noted that CStr activity in control animals appears to decrease from middle to late stages, raising concerns of data variability. This is a concern because the increased activity observed in R6/2 mice (Rebuttal Figure 2 d,e), which the authors attributed to the VIP-IN stimulation, could be at least partially due to a variation of activity across learning stages. The authors should rule out this possibility.

We showed that CStr activity increases in R6/2-ChrimsonR mice (Extended Data Fig. 8k-m). To test whether this increase is specific to R6/2-ChrimsonR mice, we now include additional analyses comparing the movement modulation across stages in all experimental groups (Ctrl-tdTomato, Ctrl-ChrimsonR, R6/2-ChrimsonR and R6/2-tdTomato).

Specifically, we tested whether the change in CStr movement modulation from middle to late stages differed across groups using a two-factor linear mixed-effects model. This analysis confirmed that R6/2-ChR mice exhibited a larger increase than the other groups ($p = 0.040$), indicating that the elevated late-stage activity cannot be explained by stage-dependent variability. We include this analysis in the updated Extended Data Fig. 8l.

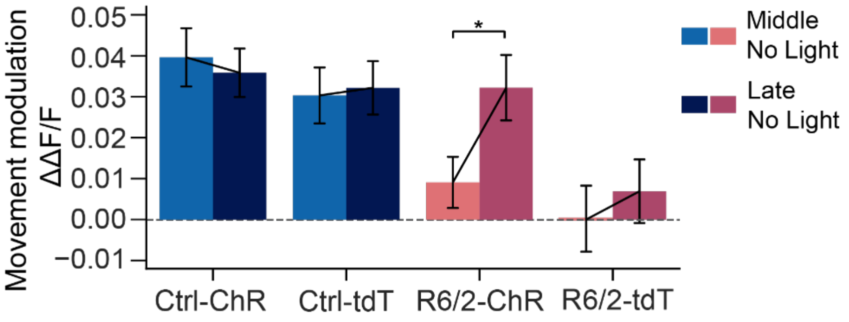

Rebuttal Figure 1 / Extended Data Fig. 8l. Mean CStr movement-related activity on no-light trials at middle and late stages. Population-level movement modulation, defined as $\Delta F/F_0$ during movement (0–8 s) minus baseline (−1–0 s), quantified across all recorded CStr neurons. R6/2-ChR mice show a larger increase from middle to late stages compared to control and R6/2-tdTomato mice. Linear mixed models with mice as random intercepts; experimental group × stage interaction (R6/2-ChR): $*p < 0.05$; effect of stage in R6/2-ChR: $*p < 0.05$; Mean ± s.e.m.

In Fig. 4, my previous concern was that there is a clear trend of decreasing stride regularity following the cessation of stimulation, which argues against the authors' claim of persistent post-stimulation effects. The authors should test this trend statistically by comparing the final stimulation session directly to the final post-stimulus session. However, the authors reported a non-significant difference by pooling the 2 sessions before and after stimulation cessation, thereby diluting this trend. If a significant decrease in stride regularity exists post-stimulation, the authors should acknowledge this result and temper their claims accordingly.

We would like to restate that our claim of persistence of rescue does not imply a complete lack of dissipation over time. We had already clearly stated this in the main text. Therefore, our claim would not be affected by whether there is a small reduction in improvements in one of the 4 behavioral measures reported.

Nevertheless, to directly address this request, we have compared the final stimulation session with the final post-stimulation session (sessions 9 vs. 11). We did not find significant differences in stimulated R6/2-ChR mice across these sessions in any of the evaluated movement parameters. Furthermore, behavioral performance in session 11 of R6/2-ChR mice remained significantly better than that in R6/2-tdT mice. We have included this analysis in Extended Data Fig. 10g and clarified this point in the Results (p. 7, lines 285-288).

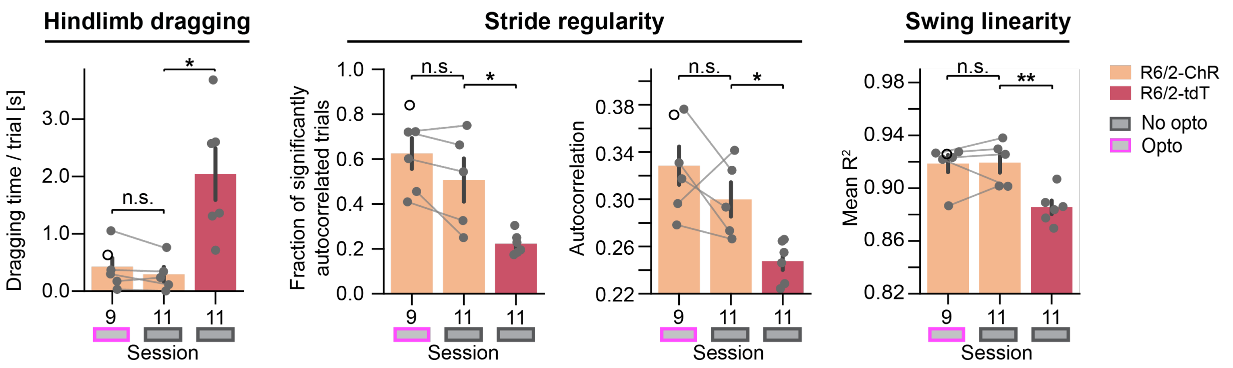

Rebuttal Figure 2 / Extended Data Fig. 10g: Stride regularity remains improved after stimulation cessation despite a visible downward trend. In R6/2-ChrimsonR mice, none of the assessed behavioral metrics – including hindlimb dragging, stride regularity and swing linearity – differed significantly between the final stimulation session (session 9) and the final post-stimulation session (session 11, paired Wilcoxon test), while all remained significantly better than R6/2-tdTomato mice at session 11 (Welch's two-sample t-test, *$p < 0.05$, **$p < 0.01$). Bars show mean ± s.e.m.; dots indicate individual mice; open circles indicate loss of one R6/2-ChR animal after session 9; grey lines connect paired measurements from the same R6/2-ChrimsonR mice.
